# Prediction of the Wingate anaerobic mechanical power outputs from a maximal incremental cardiopulmonary exercise stress test using machine-learning approach

**DOI:** 10.1371/journal.pone.0212199

**Published:** 2019-03-12

**Authors:** Efrat Leopold, Dalya Navot-Mintzer, Eyal Shargal, Sharon Tsuk, Tamir Tuller, Mickey Scheinowitz

**Affiliations:** 1 Department of Biomedical Engineering and Neufeld Cardiac Research Institute Tel-Aviv University, Ramat-Aviv, Israel; 2 The Ribstein Center for Sport Medicine Sciences and Research, The Wingate Institute, Netanya, Israel; 3 The Zinman College of Physical Education and Sport Sciences at The Wingate Institute, Netanya, Israel; US Army Research Institute of Environmental Medicine, UNITED STATES

## Abstract

The Wingate Anaerobic Test (WAnT) is a short-term maximal intensity cycle ergometer test, which provides anaerobic mechanical power output variables. Despite the physiological significance of the variables extracted from the WAnT, the test is very intense, and generally applies for athletes. Our goal, in this paper, was to develop a new approach to predict the anaerobic mechanical power outputs using maximal incremental cardiopulmonary exercise stress test (CPET). We hypothesized that maximal incremental exercise stress test hold hidden information about the anaerobic components, which can be directly translated into mechanical power outputs. We therefore designed a computational model that included aerobic variables (features), and used a new computational \ predictive algorithm, which enabled the prediction of the anaerobic mechanical power outputs. We analyzed the chosen predicted features using clustering on a network. For peak power (PP) and mean power (MP) outputs, the equations included six features and four features, respectively. The combination of these features produced a prediction model of r = 0.94 and r = 0.9, respectively, on the validation set between the real and predicted PP/MP values (P< 0.001). The newly predictive model allows the accurate prediction of the anaerobic mechanical power outputs at high accuracy. The assessment of additional tests is desired for the development of a robust application for athletes, older individuals, and/or non-healthy populations.

## Introduction

Contributions of energetic systems from aerobic metabolic pathways during exercise testing can be directly assessed using peak VO_2_ measurements [1, 2], as well as using indirect estimation of maximal VO_2_ from several metabolic equations [3, 4]. On the other hand, a direct measurement of the anaerobic capacity is considered more challenging [5] and therefore, among different available tests that measure anaerobic capacity [6–10], Medbo et al. [11] have suggested an indirect approach to measure the anaerobic capacity using the maximal accumulated oxygen deficiency (MAOD) method. The MAOD is based on estimating VO_2_ demand at supramaximal speeds by applying extrapolation, which is based on calculating the linear relationship between submaximal VO_2_ and running speed. However, the outcome of this method reflects **oxygen deficiency** as measured by blood lactate accumulation and does not reflect the “true **mechanical power outputs”** produced by the active muscles during high intensity, aerobic-type, maximal exercise stress test.

Despite the fact that the MAOD is a widely accepted tool and has been well investigated [12–14], there have been other concerns regarding this method that is being used to construct the best linear relationship, which is also time–consuming [11, 15, 16, 17]. Bangsbo [18] claimed that the MAOD method underestimates the VO_2_ demand and the accumulated O_2_ deficit during supramaximal exercise; Fletcher at al. [19] pointed out that the potential changes in substrate utilization during submaximal exercise are not considered in the prediction. Additional studies have tried to compare the MAOD with the WAnT outputs, yet correlations have been low and conclusions regarding the relationship between the variables have not been fully determined [20, 21].

During maximal incremental exercise stress test, the effort produced below the point of blood lactate accumulation (OBLA) is considered aerobic and low in terms of mechanical power output. However, above OBLA anaerobic metabolism becomes dominate and allows the generation of higher mechanical power output [22–24]. We hypothesized that maximal incremental cardiopulmonary exercise stress test (CPET) holds information of anaerobic components, which can be directly translated into mechanical power outputs (in addition to the contribution of aerobic metabolic pathways). Therefore, our goal was to predict the anaerobic mechanical power outputs from maximal incremental CPET indices using highly accurate machine-learning tools.

Prediction models have been widely used, for example, Bradshaw et al. [25] used regression analysis for the prediction of cardiorespiratory fitness level based on non-exercise data. Luttikholt et al. [26] used the critical power profile to develop a model to predict the peak power (PP) output from the outputs of various graded-exercise-stress test protocols. Their model included 11 males, and the differences between the actual and predicted PP outputs were statistically insignificant (*P* > 0.05).

The objective of our study was therefore to present a new model allowing the prediction of the anaerobic mechanical power outputs from a CPET results. The Wingate Anaerobic Test (WAnT), which has been established as an effective and accepted test to measure the anaerobic mechanical power outputs [27] was chosen for use as the gold standard in the current study.

Prediction of the anaerobic mechanical power output will provide a simpler method to estimate anaerobic mechanical power outputs using a single exercise stress test (maximal VO_2_ test). This will allow exercise physiologist, coaches, etc. to understand the contribution of the anaerobic components within aerobic exercise stress test. These data may be specially relevant for athletes who include anaerobic exercise in their routine. More specifically, knowing the mechanical power outputs would add valuable information for their training program, for example, tracking peak power can give an indication if the training program matched their goal.

## Materials and methods

### General description

**Fig 1:**
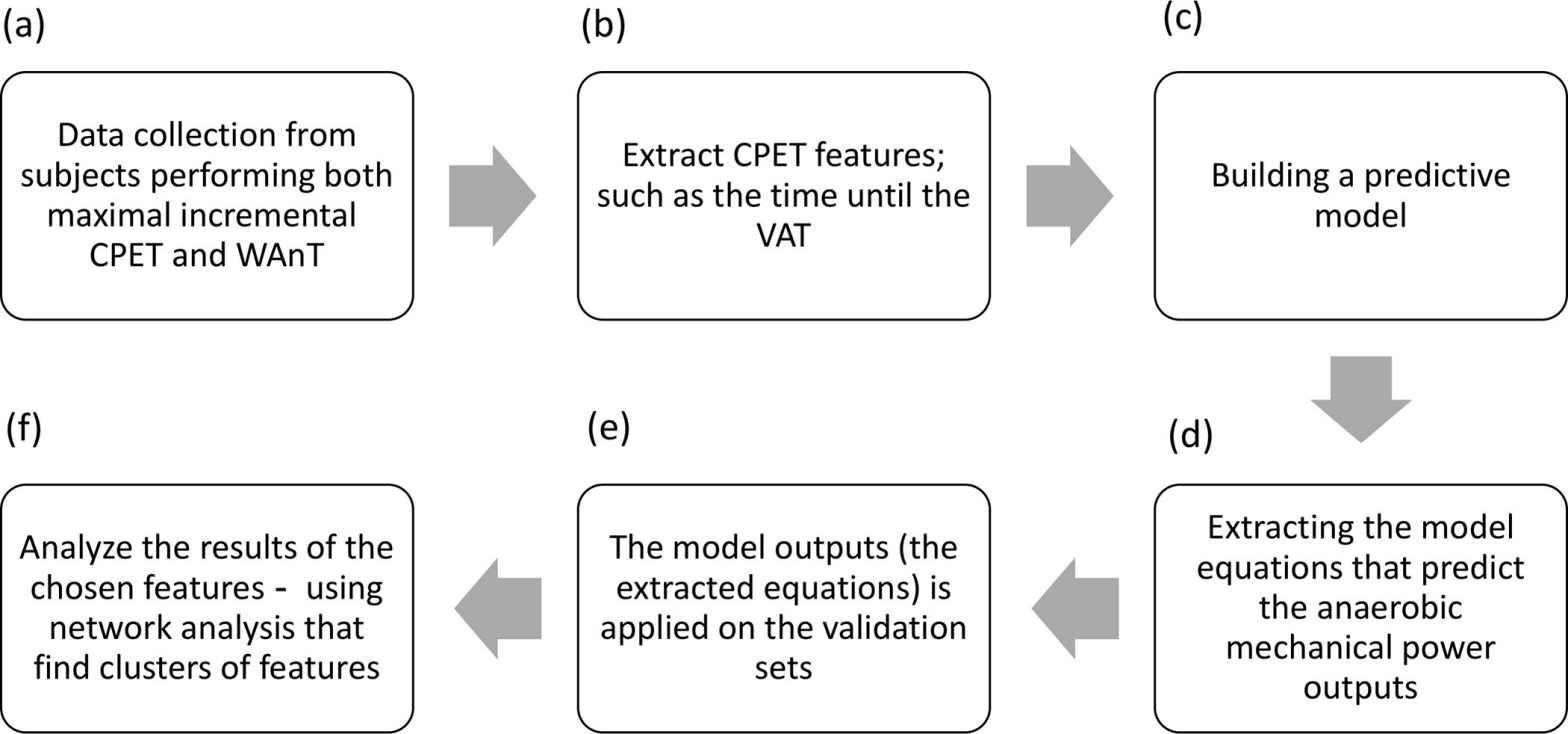
Schematic flow of the research.

A high-level predicted model was designed to predict the anaerobic mechanical power outputs based on aerobic and anaerobic exercise test results. The following steps were implemented: (1) collecting data from both maximal incremental CPET results and WAnT results (Fig 1A). (2) Extracting features of CPET result S1 Table), (Fig 1B). (3) Developing a new computational model from maximal incremental CPET results to facilitate the prediction of the anaerobic mechanical power outputs (peak power, PP; mean power, MP; fatigue, %) (Fig 1C). This computational process was performed successfully in previous studies [28–31]. (4) Using the outputs of the model (linear regression mathematical equations) and apply it on the validation sets (Fig 1D and 1E). (5) Using network analysis to interpret the results of the predicted model (Fig 1F). A detailed illustration of the flow of the study is shown in Fig 1.

### Data collection

The study was approved by the local IRB Committee at Hillel Yaffe Medical Center, approval number 0035-14-HYMC. Normal, healthy male and female, athletes and non-athletes signed an informed consent form and thereafter performed both the WAnT and the CPET tests. They either studied at the Zinman College for Physical Education or have been tested at the Department of Research and Sports Medicine, Wingate Institute, Israel.

### Wingate test and CPET

The WAnT was used to measure the anaerobic mechanical power outputs; PP and MP [32]. All participants (n = 88) were instructed to perform a warm up on a cycle ergometer for 3 minutes at moderate intensity. Following the warm-up period, the cycle ergometer was programmed for a 30-s test duration against resistance equals to 0.075 of the body weight [27]. The participants then completed an “all-out” 30-s effort with verbal encouragement. PP, MP, and fatigue index were calculated and then displayed on the ergometer screen at the end of the test. PP was described as the maximal mechanical power output attained during the first few seconds from the beginning of the test, and MP was described as the average power over the entire 30 s of the test. The fatigue was calculated as the percent difference between maximal mechanical power output and minimal mechanical power output achieved during the test [33].

Approximately two weeks after performing the WAnT, each participant underwent a running maximal incremental CPET. Information regarding the test has been provided to each subject prior to the test. The Quark CPET (COSMED, Rome, Italy) was used, and calibration was performed using the ERGO-RMR and TURBINE software at the beginning of each day of the trial. The machine was recalibrated if needed. The test started with a short warm-up period of 2–3 minutes at low walking speeds. The protocol included increasing the speed gradually by 1 [kilometer/hour] every minute, and when the respiratory quotient reached 1, the slope was increased by 2%. The test ended when the subject’s reached his/her maximum heart rate, as measured by a Polar watch or ECG. Ventilatory and metabolic variables such as VO_2_, VCO_2,_ and VE were recorded breath-by-breath during the entire test until the subjects reached maximal effort.

### Algorithm to predict the anaerobic mechanical power output

We developed a greedy heuristic algorithm to study the ability of aerobic features to predict the anaerobic mechanical power outputs. This algorithm uses a locally optimal choice of features at each iteration, and is described below:

#### Feature generation

The features represent the aerobic variables that are measured directly during the CPET, as well as additional parameters that can be extracted and calculated from the measured parameters. The features were classified into two different sets: (1) features that are being measured directly, such as duration of the test [s], minute ventilation [l/min], oxygen consumption [ml/min], etc., and (2) features that are calculated and derived from the directly measured parameters, such as VE/VO_2_, VE/VCO_2_, slope of the VCO_2_ versus VE graph, VE at the ventilatory anaerobic threshold (VAT, which was determine via the V slope method), etc.

Overall, there were 12 directly measurable features and 39 calculated features (S1 Table), taken from the entire duration of maximal incremental CPET. These features were extracted under the assumption of their impact on the mechanical power outputs and as such, for example, the features related to the VAT, depends on numerous physiological parameters, such as VO_2_max and VE max, which can potentially contribute to the anaerobic power outputs prediction [34].

In order to understand the ability to predict the mechanical power outputs from early stages of maximal incremental CPET, further features were generated which represent the first to four minutes of the maximal incremental CPET. Such features included, for example, the slope of VO_2_ versus time (up to the first minute) and the VE calculated for each minute.

We then divide the features into 2 subgroups: (1) features taken from the entire duration of maximal incremental CPET, and (2) features from the early stages of maximal incremental CPET. Features related to the subjects such as body weight, height, and body mass index were not included in our predicting model.

#### Description of the feature selection procedure

**Fig 2:**
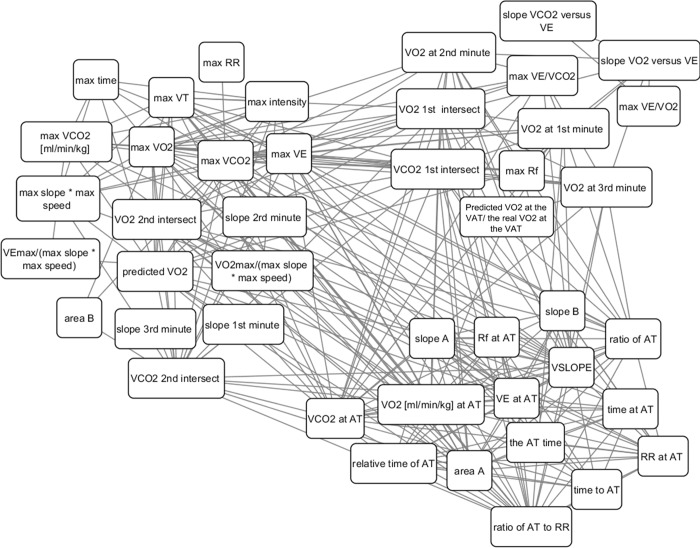
Flow diagram that describe the feature selection procedure.

The flow chart of the feature selection procedure is shown in Fig 2. First, we created a feature matrix that included all features. All features were standardized so that at each column the mean and standard deviation (SD) were 0 and 1, respectively. The features were divided into three random groups: 40% of the data were assigned to the train subset, 30% of the data were assigned to the test subset (both of these data sets represent the calibration set), while 30% of the data were used for the validation set.

The algorithm automatically selected the ‘best’ feature (i.e. the feature that demonstrates the smallest distance between the predicted to the observed values of the anaerobic mechanical power output; see below) at each iteration and added it to the vector of selected features. In order to ensure uniform selection of the range (from all exercise levels) for the train and test subsets within the calibration set, VO_2_ max was divided into three groups, and at each iteration two random subsets were selected from these groups. The following steps were then applied:

Using the train subset:

1. Selecting the *i*-th (i = numbers of features) feature from the aerobic features matrix.
2. Performing multiple linear regressions and using the coefficients obtained to calculate the predicted anaerobic parameter (each at a time).
3. Checking and saving the prediction error, which is the distance between the predicted values obtained from the updated predictor to the observed value of the anaerobic parameter (using the least squares method).
4. Performing steps 1–3 on all features, and adding the feature with the smallest distance between the predicted values obtained from the updated predictor to the observed value of the anaerobic parameter.
5. Adding another feature (i+1) and repeating steps 1–4.
6. Continuously adding features until the termination condition is encountered (see below).

Using the test subset:

1. The selected feature and chosen coefficients were used to calculate the predicted anaerobic parameter (each at a time).
2. Calculating the Spearman correlation coefficient obtained from the predicted anaerobic parameter compared with the real value.
3. The loop is terminated according to the percentage change in the adjusted R^2^. If the adjusted R^2^ of the added predictor is 2% smaller than the R^2^ adjusted of the current predictor, the algorithm stops adding features to the predictor.

The algorithm output is a linear regression mathematical equation that represents the best-chosen features, together with the features’ coefficients, which is also referred as the predictor (described by Palmer and O'Connell [28]):

Anaerobicmechanicalpoweroutput=α+β*feature1+γ*feature2+δ*feature3… (1)

○ The random division of the data may create different combinations of the chosen features. Therefore, to ensure that the most common features were selected, the algorithm was run 1000 times using the new data set each time.
○ The equation extracted for each of the anaerobic mechanical power output parameter was then applied to the validation set.
○ The aforementioned procedure was performed 100 times, at which each time the data was randomly divided into train, test and validation groups.
○ The results are presented using Spearman correlation coefficients of the predicted anaerobic parameter versus the real value and the SD of the correlations.
○ The mean and SD of the percent error between the predicted anaerobic parameters with the real values are also reported.

### Clustering analysis of the features based on a network representation

The network used in this study contains features that are related to the respiratory, cardiovascular and metabolic systems. Accordingly, in order to characterize the structure of this network, the modularity and community structure in networks as defined by Newman was used [35]. That is, this was carried out in order to explore the connectivity between the aerobic features, and to determine whether there are any natural divisions of aerobic features into groups (for which these groups may be of any size). Q is the modularity and is equal to:

$$Q=\frac{1}{4m}\sum_{ij}\left(Aij-\frac{ki*kj}{2m})(SiSj+1\right)$$ (2)

Where Aij is defined as the number of edges between vertices i and j. If the edges are placed at random, the expected number of edges between vertices i and j is equal to: *kikj*/2*m*

Where *ki* and *kj* are the degrees of the vertices and *m* is the total number of edges in a network. The aim is to maximize the modularity by choosing a correct division of the network, which is manifested in the value of the index vector **S**.

In order to divide the networks into more than two groups, the network was initially split into two groups, and these were then iteratively divided each into two, and so on.

$$△Q=1/2m[\frac{1}{2}\sum_{ij∊g}Bij(SiSj+1)-\sum_{ij∊g}Bij$$ (3)

The goal was to maximize △*Q*.

**Fig 3:**
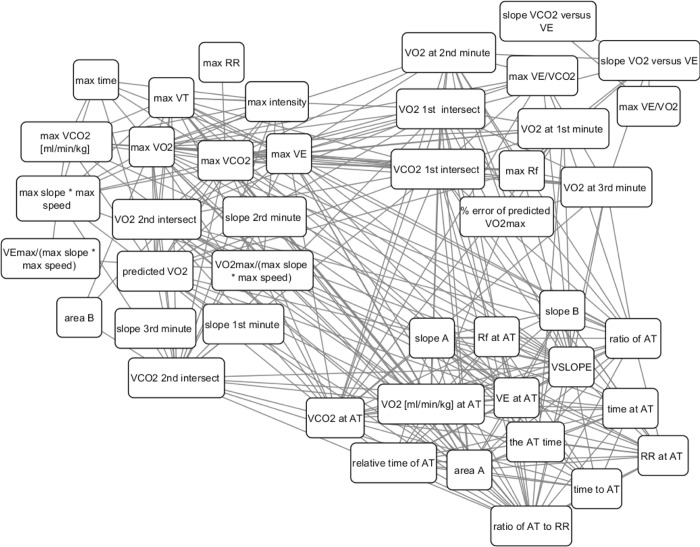
Representation of a network that connects all the aerobic features.

The network included the aerobic features, which were extracted from the CPET results (VO_2_ max, VCO_2_ max, etc.). Overall, there were 51 features for which the Spearman correlation coefficients were calculated. These were incorporated into a 51*51 matrix. Each aerobic feature was represented by a node, and its Spearman correlation coefficients with any other aerobic feature was represented by an edge (vertex). Fig 3 shows a representation of a network that connects variables. The weight of the vertex is equal to the coefficient of the Spearman correlation between the two relevant features.

The aforementioned algorithm was applied to the 30% of the most highly correlated features within the aerobic matrix in order to identify any natural divisions of its vertices into groups.

### Statistical analysis

Anthropometric and exercise data are presented as mean ± SD. The model was applied to 100 randomly selected validation sets in order to determine the SD. The mean ± SD of the percent error was also calculated. A *p* ≤ 0.05 was considered statistically significant.

## Results

### Experimental data

General characteristics, as well as the aerobic and anaerobic outputs of the male and female participants are presented in Table 1.

**Table 1:** General characteristics as well as CPET aerobic and WAnT anaerobic outputs of the participants in the study.

Variable	Mean± SD	Mean± SD
	Female (N = 36)	Male (N = 52)
Age	25 ± 4	28 ± 6
Body Height (cm)	164.3 ± 6.4	176.6 ± 6.8
Body Mass (kg)	60.7 ± 8.7	75.8 ± 10.2
Body Mass Index	22.5 ± 2.8	24.3 ± 2.7
Maximal Oxygen Consumption (ml/min)	2585.9 ± 382.7	4169.2 ± 605.8
Maximal Minute Ventilation (l/min)	87.2 ± 14.5	146.5 ± 20.6
Maximal Heart rate (beats/min)	183 ± 7.8	186 ± 7.3
Peak Power (w)	450.8 ± 84.7	767.1 ± 127.8
Mean Power (w)	333.3 ± 62	573 ± 101.5
Fatigue Index (%)	49.5 ± 15.1	50.1 ± 9.3

### Model predictions

By applying the algorithm to features from all stages of the maximal incremental CPET, the anaerobic PP [w] and MP [w] prediction equations derived from the validation sets are presented in Table 2. For PP [w], the equation includes six features and the combination of these features produced a Spearman correlation coefficient of 0.94 and SD of 0.1, between the predicted and real PP [w] values of the validation sets. For the MP [w] predictions, four features were automatically chosen and the combination of these features produced a Spearman correlation coefficient of 0.9 and SD of 0.07 between the predicted and real MP[w] values of the validation set. The data of the selected features is found in S2 Table (and raw data of the features is found in S3 Table).

**Table 2:** Multiple linear regression equations, together with the Spearman correlation coefficient and P-value, of the predicted equations for the validation group, for the peak power (PP) and mean power (MP).

Category	Multiply regression equation	R^2^ and SD	RMSE	Mean ± SD of % error
Peak Power (w)	638.4 + max VE * 170.3—max RF * 43.5—max VO2 * 77.5 + max slope * max speed * 98.6 + slop VCO2 versus VE * 39.3 + VO2max/(max slope * max speed) * 53	0.94 (P = 2.1*e^-06^), SD = 0.1	96	19 ± 18
Mean Power (w)	476.8 + max VE * 105.5 + slope 1^st^ min of VO2 vs. time * 36.4—max VO2 * 33.8 + max slope * max speed * 27.6	0.9 (P = 2.7*e^-06^), SD = 0.07	73	16 ± 14

Anaerobic power outputs had higher Spearman correlations coefficient with equation predictions than aerobic variables. For example, the highest Spearman correlation coefficient between PP [w] and the maximal value of VE was 0.81, compared with the prediction Spearman correlation coefficient of 0.94 resultant from a combination of 6 features (Table 2).

**Fig 4:**
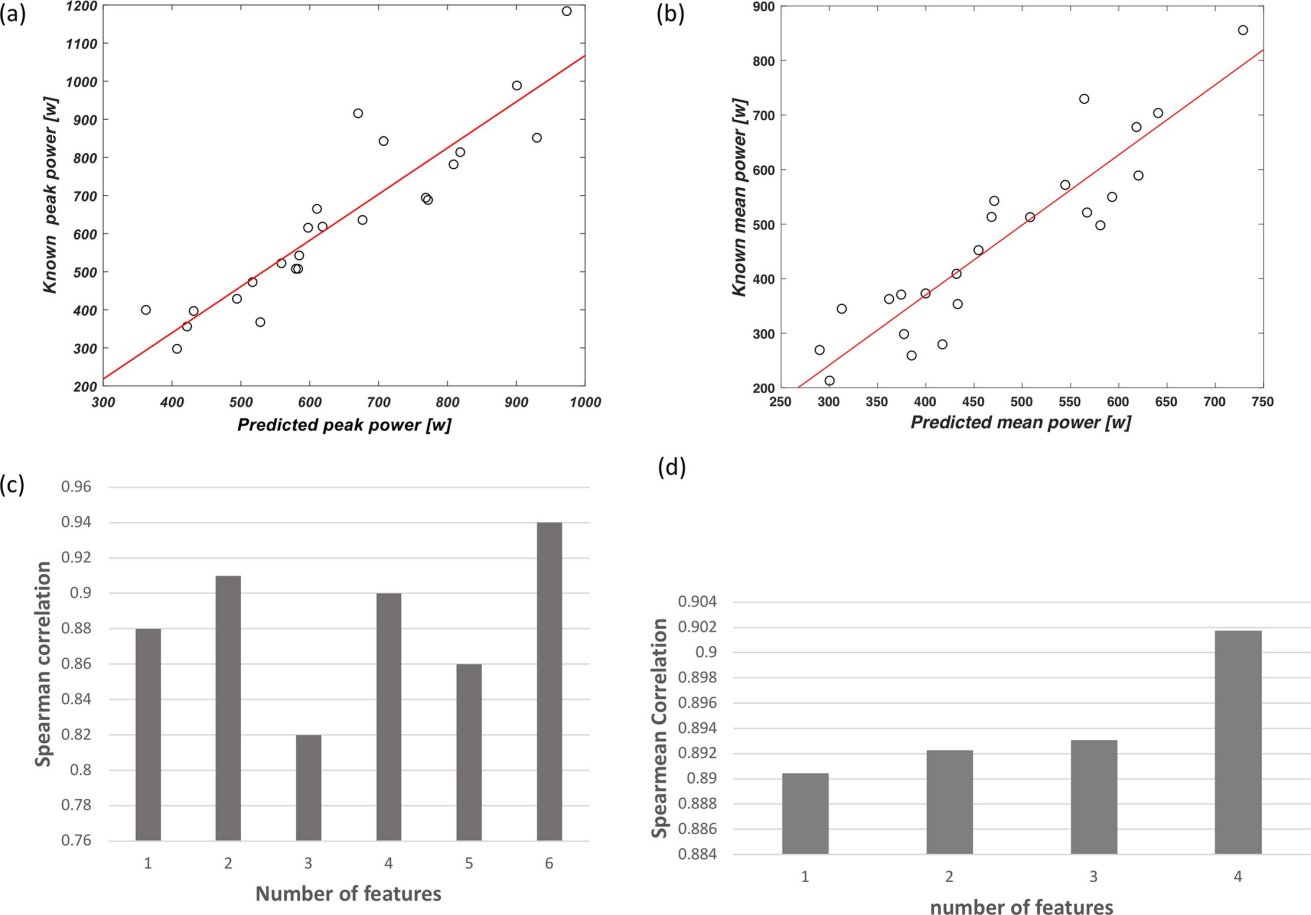
Plots illustrating the validation set for the predicted (x-axes) versus the known (y-axes) values for peak power (PP) (w) (a) and mean power (MP) (w) (b). The bar diagram illustrates the Spearman correlation coefficient between the predicted values of the peak power (PP) (w) (c) and mean power (MP) (w) (d) while adding an additional feature each iteration.

Fig 4 shows dot plots and a bar diagram of the validation set, as derived from the equations described in Table 2. The plots illustrate the known versus the predicted values of the PP [w] (a) and MP [w] (Fig 4B). The bar diagrams illustrate the correlations between the predicted values of the PP[w] (a) and MP [w] (b), while adding an additional feature each iteration. More specifically, for PP the additional features increase and decrease the prediction, up until reaching the sixth feature, at which the prediction reaches 0.94 (p–value = 2.7*e^-06^). For MP, the additional feature at each iteration increase the prediction, at which the fourth feature produce prediction of 0.9 (p-value = 2.7*e^-06^).

**Fig 5:**
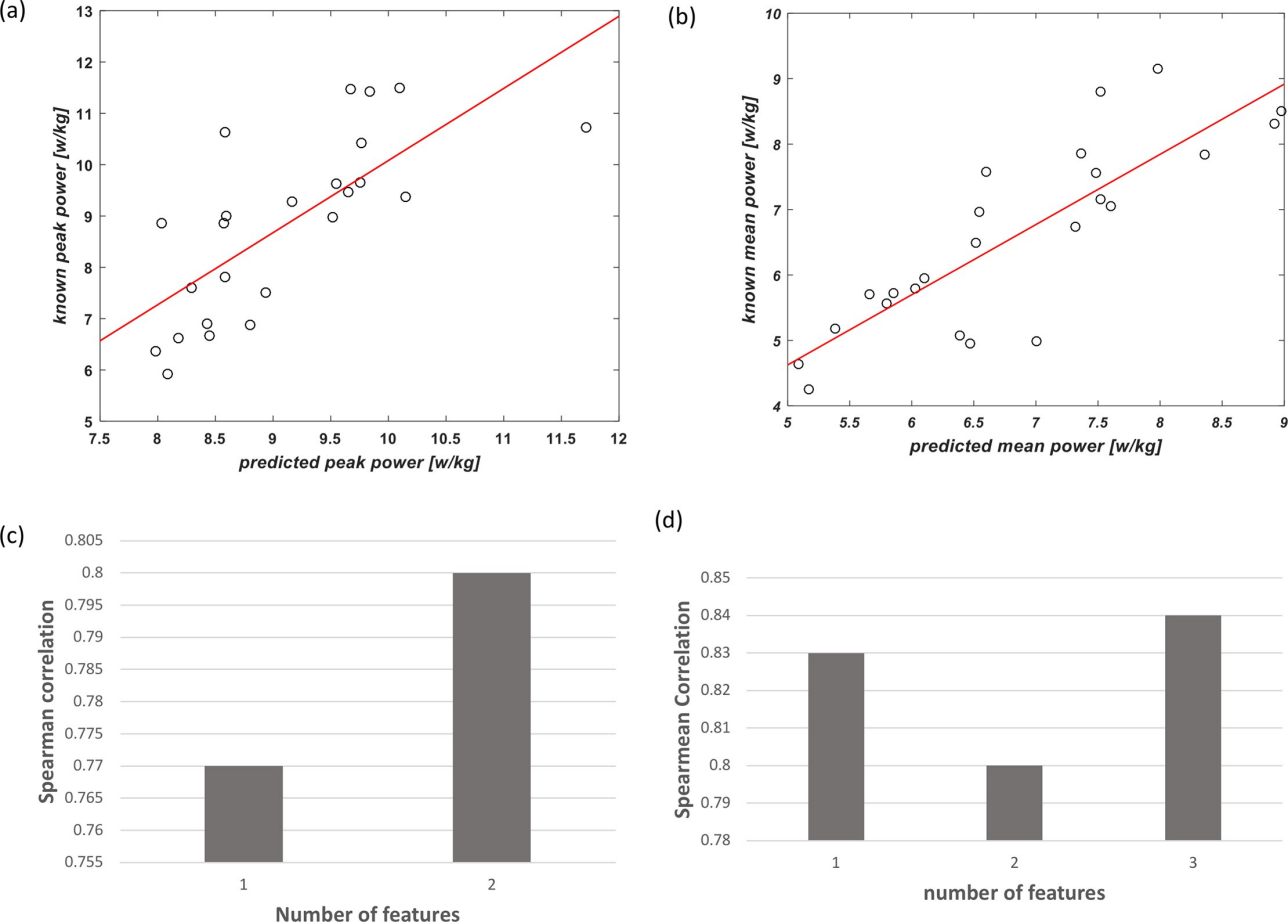
Plots illustrating the validation set for the predicted (x-axes) versus the known (y-axes) values for peak power (PP) (w/kg) (a) and mean power (MP) (w/kg) (b). The bar diagram illustrates the Spearman correlation coefficient between the predicted values of the peak power (PP) (w/kg) (c) and mean power (MP) (w/kg) (d) while adding an additional feature each iteration.

By looking at the anaerobic PP and MP normalized by body weight [w/kg], and by applying the algorithm, the prediction of mechanical power outputs derived from the validation set are presented in Fig 5. The combination of features produced a Spearman correlation coefficient of 0.84 and 0.8, between the predicted and real PP [w/kg] and MP [w/kg] values of the validation set, respectively.

### Model predictions based on the first few minutes

The model generated based on features from the first to fourth minutes exhibited lower prediction of the anaerobic mechanical outputs compared with the model generated based on features from the entire duration of the test. Specifically, the Spearman correlation coefficient between the prediction and measurements for the first few minutes-based model was ~ r = 0.77 (p-value = 8.1*e^-05^) compared with r = 0.94 (p-value = 2.1*e^-06^) for the whole set-based model for PP and ~ r = 0.78 (p-value = 2.1*e^-05^) compared with r = 0.9 (p-value = 2.7*e^-06^) for the whole set-based model for MP.

### The clustering/network

In order to analyze the chosen features from the predictive model, a network analysis was performed to identify clusters of features with similar statistical power.

Table 3 shows the outputs of the 3 groups from the clustering network algorithm. Each group describes a cluster of features that has greater strength of relationship than with the features from the other groups. Group 1 includes two subgroups: the directed measurable parameters such as maximal VE, and calculated features related to the intensity of the subjects such as maximal duration of the test, maximal slope, etc. Group 2 represents features related to the VAT, as well as to the parameters related to the VCO_2_ versus VO_2_ graph, such as the slope up to the VAT. Group 3 includes features related to the VAT, as well as relative features related to the duration of the test.

**Table 3:** The clustering of features based on a network.

Group 1	Group 2	Group 3
max time	time to reach VAT	max VE/VO_2_
max VT	time from the VAT to the end	max VE/VCO_2_
**max VE (PP,MP)**	relative time of VAT	VO_2_-1 at VAT
max VO_2_	ratio of VAT (VO_2_) to the max time	VCO_2_-1 at VAT
max VCO_2_	ratio of VAT to the max value of RER(VO_2_)	**slope VCO**_**2**_ **versus VE (PP)**
**max VO**_**2**_ **[ml/min/kg] (PP,MP)**	Slope A	slope VO_2_ versus VE
max RR	Slope B	VO_2_ at 1^st^ min
VO_2_-2 at VAT	area A of graph VO_2_ as a function of VCO_2_	VO_2_ at 2^nd^ min
VCO_2_-2 at VAT	Time at VAT	VO_2_ at 3^rd^ min
area B of graph VO_2_ as a function of VCO_2_	RF at VAT	predicted VO_2_ at VAT/the real VO_2_ at VAT
**slope at 1st min increase VO**_**2**_ **(MP)**	VE at VAT	**max RF (PP) **
slope at 2nd min increase VO_2_	VCO_2_ [ml/min] at VAT	
slope at 3rd min increase VO_2_	VO_2_[ml/min/kg] at VAT	
**max slope time max speed (PP, MP)**	RR at VAT	
VEmax/slope*speed	VSLOPE	
**VO**_**2**_max/slope*speed (PP)		
predicted VO_2_		
max slope * max speed * max time		

## Discussion

The present study demonstrates a new predictive model for the assessment of anaerobic mechanical power outputs (as an outcome obtained from the WAnT) using maximal incremental CPET results. This research is a proof of concept, with aim of suggesting a method to perform only aerobic exercise stress test and to use aerobic features, from a single test, to identify the anaerobic mechanical power outputs. Our study demonstrates an array of multiple aerobic features with constant weight that accurately predict the PP and MP mechanical power outputs, as shown below:

$$PP\;[w]\;prediction=638.4+170.3*\max\;VE-43.5*Rf-77.5*VO2max+98.6*\max\;slope*\max\;speed+39.3*slope\;VCO2\;vs.VE+53*\frac{VO2max}{\max\;slope*\max\;speed}$$ (4)

MP[w]prediction=476.8+105.5*maxVE+36.4*slope1stminofVO2vs.time+33.8*VO2max+27.6*maxslope*maxspeed (5)

For PP prediction, the chosen features relate to the intensity of the exercise stress test through features that include the maximal slope and the maximal treadmill speed, as well as features that relate to the subject’s aerobic capacity, such as VO_2_max and maximal VE. The rational behind the chosen features can be explained and here we chose to explain two features: VO_2_max and the slope of the VE vs. VCO_2_ graph. During high-level exercise, higher VO_2_max levels means that more adenosine triphosphate (ATP) energy can be produced. Beneke et al. [36] estimated the fraction of the aerobic contribution of the WAnT to be approximately 18.6%, indicating a significant contribution of the oxidative systems in this short duration (30 sec) and intense exercise. Therefore, the higher the VO_2_max is, the more likely that the subject engages in regular aerobic exercise, and the cardiovascular system therefore adapts to that exercise. These adaptations then transfer into greater performance during high-intensity exercise (such as the PP) [33–34], thus, VO_2_max serve as important features that contribute to the prediction of PP. The slope of the VE vs. VCO_2_ graph, has been found to be significantly correlated with decreased cardiac output, elevated pulmonary pressures and associated with increased ventilation-perfusion mismatching [37], and therefore might serve as a feature indicative of the subject’s ability to attain higher/lower PP values.

For MP predictions, the rational behind the chosen features can also be explained, as it reflects the ability of the subjects to withstand the intense physical exercise during the 30 s WAnT test. One example, is the slope of the 1^st^ minute of the VO_2_ vs. time graph which represents the ‘O_2_ deficit’ proposed by Medbo et al. [11]. This O_2_ deficit, which is the difference between the amount of energy that is required to perform a certain work rate (power output) exercise and the amount of energy that is supplemented through oxidative metabolism, must be supplied through anaerobic pathways to compensate this lack. Therefore, a reduced oxygen deficit would improve exercise performance, as it reduces the usage of muscle anaerobic metabolites such as phospho-creatine and glycogen [38], hence, this feature can indicate on the ability of a subject to efficiently use the anaerobic pathways which is related to MP, and therefore to the ability to predict it.

All the features are connected with each other as shown in Table 3. In each group there are features that tend to change in a correlative manner among the analyzed subjects. This means that we expect that features from the same cluster can be replaced other features from the same cluster in our predictor and give relatively similar predictions (for example max VE has high correlation of 0.88 with max VO2 and they both appear in the first group). This means that there are more than one good predictors with similar performances and can help understanding the physiological meaning of the predictors via looking at features that are correlative (similar) with the predictor's features.

This research has further demonstrated that features extracted from the entire test predict the anaerobic mechanical power outputs better than features from only the early CPET stages. Furthermore, predictions of PP and MP, when normalized to body weight, showed lower correlations than the absolute values of PP and MP (r = 0.94 versus 0.84 and 0.91 versus 0.8, respectively), as shown in Figs 4 and 5. This study, however, excluded the prediction of fatigue [%] since it was low and not significant (r = 0.4 p value > 0.05).

To summarize, the ability to predict the anaerobic mechanical power outputs will allow exercise physiologist, coaches, etc. to understand the contribution of the anaerobic components within a single aerobic exercise stress test. The WAnT results, which are the outputs of the predictive algorithm, reflect the changes in the contribution of the anaerobic energy systems to the performed exercise [27, 39, 40]. This may be relevant, for instance, for athletes and coaches that by knowing if the subject utilize glycogen or free fatty acid, can help specify the exercise program. Another example of the usefulness of the model’s outputs is that knowing the PP of a subject gives insight regarding the type of muscle fibers (fast/slow) that the subject has, since high or low values of PP are observed in subjects who probably have high or low proportions of fast twitch fibers, respectively [41]. This information will therefore help trainers to provide better exercise recommendations.

When drawing conclusions from the results of our study, one must consider the study population, which included healthy males and females within age range of 20–40 years old. It is desirable to develop a test applicable to older and/or non-healthy populations as well; future research in this field is therefore required. Moreover, analysis on individuals undergoing repeat tests should also be conducted in order to investigate changes that might occur over training/time. Additional limitations of this study are originated in the nature of the algorithm, according to which the chosen predictable features might change for different datasets. Furthermore, changing the termination condition can affect the number of the chosen predictable features.

Overall, we have presented a new concept that allows exercise specialists (physiologist and others), when conducting maximal incremental CPET, to generate additional valuable information regarding anaerobic mechanical power outputs which can affect exercise recommendation outcomes.

## Supporting information

S1 Table Features descriptions. (PDF)

S2 Table Data of the selected features. (PDF)

S3 Table Raw data of all features. (PDF)
