# Supplementary material for: Prediction of the Wingate anaerobic mechanical power outputs from a maximal incremental cardiopulmonary exercise stress test using machine-learning approach
Source: PLoS One. 2019 Mar 12;14(3):e0212199. doi: 10.1371/journal.pone.0212199 (PMC6413913; doi:10.1371/journal.pone.0212199)
Supplement: S1 Table — (PDF) [file pone.0212199.s001.pdf]

Table A in S1 File. Features descriptions

Features (N=51)	Description
Max time [s]	Maximal duration of the test
Max RF [1/min]	Respiratory frequency which refers to the breathing rate per minute
Max VT [L]	Maximal tidal volume. This is the lung volume that represents the normal volume of air displaced between normal inhalation and exhalation
Max VE [L/min]	VE refers to minute ventilation. This is calculated by multiplying tidal volume by respiratory frequency
Max VO_2 [mL/min]	Maximal volume of oxygen consumption (VO_2) during the test
Max VCO_2 [mL/min]	Maximal volume of carbon dioxide production (VCO_2) during the test
Max VO_2 [mL/min/Kg]	Maximal volume of oxygen consumption during the test, as measured in milliliters per kilogram of body weight per minute
Max HR [beat/min]	Maximal heart rate
Max RR	The respiratory quotient, is a dimensionless number calculated from the ratio of carbon dioxide produced by the body to oxygen consumed by the body
Max VE/VO_2	Maximal minute ventilation divided by maximal volume of oxygen consumption
Max VE/VCO_2	Maximal minute ventilation divided by maximal volume of carbon dioxide production
VO2 1st intersect [mL/min]	Oxygen consumption value of the 1st intersect when fitting three linear lines to the carbon dioxide production as a function of oxygen consumption
VO2 2nd intersect [mL/min]	Oxygen consumption value of the 2nd intersect when fitting three linear lines to the carbon dioxide production as a function of oxygen consumption
VCO2 1st intersect [mL/min]	Carbon dioxide production value of the 1st intersect when fitting three linear lines to the carbon dioxide production as a function of oxygen consumption
VCO2 2nd intersect [mL/min]	Carbon dioxide production value of the 2nd intersect when fitting three linear lines to the

	carbon dioxide production as a function of oxygen consumption
V SLOP [mL/min]	The ventilatory anaerobic threshold determined using the V slope method
Time to the VAT [s]	The time it takes to reach the ventilatory anaerobic threshold
Relative aerobic time	The time it takes to reach the ventilatory anaerobic threshold divided by the duration of the test
The anaerobic time [s]	The time from the ventilatory anaerobic threshold to the end of the test
Relative time of VAT	The time from the ventilatory anaerobic threshold to the end of the test divided by the duration of the test
VAT ratio	Oxygen consumption value at the ventilatory anaerobic threshold divided by the duration of the test
Ratio of VAT to RR	Oxygen consumption value at the ventilatory anaerobic threshold divided by the maximal value of the respiratory quotient
Slope VCO_2 versus VE	Slope in the volume of carbon dioxide production as a function of minute ventilation graph
Slope VO_2 versus VE	Slope in the oxygen consumption volume as a function of VE graph
Slope VO_2 versus time	Slope in the oxygen consumption volume as a function of time graph
Slope A	Slope of the volume of carbon dioxide production as a function of oxygen consumption volume graph up to the ventilatory anaerobic threshold
Slope B	Slope of the volume of carbon dioxide production as a function of oxygen consumption volume graph from the ventilatory anaerobic threshold to the end of the test
Area A	Area of the volume of carbon dioxide production as a function of oxygen consumption volume graph up to the ventilatory anaerobic threshold
Area B	Area of the volume of carbon dioxide production as a function of oxygen consumption volume graph from the ventilatory anaerobic threshold to the end of the test
Slope RR versus time	Slope of the respiratory quotient as a function of time graph
Slope VT as a function of time	Slope of the tidal volume as a function of time graph
Slope 1st minute	Slope of the 1st minute in the oxygen consumption volume as a function of time graph

VO_2 at 1st minute [mL/min]	Oxygen consumption value at the 1st minute in the oxygen consumption volume as a function of time graph
Slope 2nd minute	Slope of the 2nd minute in the oxygen consumption volume as a function of time graph
VO_2 at 2nd minute [mL/min]	Oxygen consumption value at the 2nd minute in the oxygen consumption volume as a function of time graph
Slope 3rd minute	Slope of the 3rd minute in the oxygen consumption volume as a function of time graph
VO_2 at 3rd minute [mL/min]	Oxygen consumption value at the 3rd minute in the oxygen consumption as a function of time graph
Predicted VO_2 / the real VO_2	The predicted maximal oxygen consumption value divided by the real value of the maximal oxygen consumption
Predicted VO_2 at the VAT/ the real VO_2 at the VAT	The predicted maximal oxygen consumption value at the ventilatory anaerobic threshold divided by the real value of the maximal oxygen consumption at the ventilatory anaerobic threshold
Time at the VAT [s]	The time at the ventilatory anaerobic threshold
RF at the VAT [1/min]	Respiratory frequency at the ventilatory anaerobic threshold
VT at the VAT [L]	Tidal volume at the ventilatory anaerobic threshold
VE at the VAT [L/min]	Minute ventilation at the ventilatory anaerobic threshold
VCO_2 at the VAT [mL/min]	Value of the carbon dioxide production at the ventilatory anaerobic threshold
VO_2 at the VAT [mL/min/Kg]	Oxygen consumption value at the ventilatory anaerobic threshold
RR at VAT	The respiratory quotient at the ventilatory anaerobic threshold
Max slope * max speed	Maximal value of slope multiplies by the maximal value of speed
VEmax/(max slope * speed)	Maximal minute ventilation divided by the maximal value of slope multiplies by the maximal value of speed
VO_2max/(max slope * speed)	Maximal value of oxygen consumption divided by the maximal value of slope multiplies by the maximal value of speed
Predicted VO_2 max [mL/min/Kg]	Predicted maximal oxygen consumption based on ACSM equation: VO_2 = 0.2 *(speed) + 0.9 (speed)*(fractional grade) + 3.5

Max intensity	Maximal value of slope multiplied by the maximal value of speed multiplied by the duration of the test
