# Supplementary material for: Prediction of the Wingate anaerobic mechanical power outputs from a maximal incremental cardiopulmonary exercise stress test using machine-learning approach
Source: PLoS One. 2019 Mar 12;14(3):e0212199. doi: 10.1371/journal.pone.0212199 (PMC6413913; doi:10.1371/journal.pone.0212199)
Supplement: S2 Table — (PDF) [file pone.0212199.s002.pdf]

Table B in S2 File. Data of the selected features

Subject #	Max RF [1/min]	Max VO_2 [mL/min/Kg]	Max VE [L/min]	Slope VCO_2 versus VE	Slope 1st minute	Max slope * max speed	VO_2max / (maximal slope * speed)	Peak power (w)	Mean power (w)
1	62.5	57.7	156.7	29.9	22.2	130	31.9	803.8	583.2
2	43.0	58.8	126.4	42.8	13.2	100	39.8	768.4	528.1
3	49.9	54.1	131.9	37.8	25.5	130	29.9	853.9	613.2
4	59.1	54.4	151.5	31.6	17.3	99	40.7	687.2	497.9
5	57.0	43.8	104.6	32.9	6.7	70	41.1	527.8	428.4
6	55.2	34.9	90.9	32.1	9.4	88	26.6	477.2	344.4
7	43.7	39.7	91.1	32.1	5.9	90	25.6	472.5	359.6
8	51.7	48.6	80.9	35.9	20.1	72	35.1	357.3	259.2
9	43.2	41.3	92.2	34.9	11.7	40	69.3	477.9	311.4
10	46.2	49.1	96.2	34.5	-9.7	130	24.5	775.2	576.0
11	35.6	38.2	54.7	42.4	4.9	64	31.1	395.4	269.3
12	72.3	51.4	167.3	27.4	16.8	120	33.2	833.5	673.1
13	42.9	53.5	93.0	38.9	18.0	110	28.5	543.2	409.0
14	47.1	51.8	160.3	38.3	49.7	104	51.3	1184.1	855.8
15	46.0	33.2	80.5	36.8	7.5	48	54.2	508.7	370.8
16	49.1	55.5	123.1	35.7	22.0	120	32.9	801.9	607.9
17	39.4	43.2	82.2	41.4	20.9	55	49.1	626.7	478.7
18	50.3	44.4	83.8	34.7	9.2	66	36.3	477.3	352.1
19	42.1	46.6	79.6	34.0	-2.0	70	41.2	427.5	344.8
20	35.8	42.6	45.1	47.5	1.8	60	35.5	473.8	328.6
21	52.6	44.5	151.0	34.0	9.6	110	36.4	975.7	772.7
22	46.0	47.7	90.9	41.0	15.4	90	28.3	406.7	316.3
23	55.2	46.7	110.9	34.3	6.7	95	31.9	618.5	452.4
24	33.7	53.5	71.7	41.3	2.7	70	35.5	414.1	306.4
25	46.2	41.4	79.9	39.4	6.2	72	36.5	473.4	362.6
26	57.0	69.4	147.7	33.9	18.2	112	39.4	664.0	517.1
27	58.8	48.2	94.4	37.3	1.5	66	44.6	525.0	416.1
28	48.5	38.5	70.4	33.9	-2.0	54	35.6	296.3	213.0
29	47.2	39.6	80.9	35.6	6.9	72	35.2	442.2	329.4
30	72.7	42.0	181.9	23.9	16.9	104	34.9	892.6	697.8
31	43.8	43.1	97.0	31.1	5.5	70	39.6	507.1	372.9
32	45.3	46.4	154.2	32.4	18.4	96	45.8	852.6	678.1
33	48.9	38.8	96.6	26.9	9.3	60	36.2	367.0	279.6
34	52.2	51.2	106.0	34.7	18.6	99	33.8	614.9	513.3
35	54.1	54.9	153.9	29.7	6.0	132	31.6	851.6	653.7
36	44.8	41.1	132.1	29.1	16.1	80	41.4	953.9	714.8
37	59.2	61.0	146.8	28.4	26.8	90	45.1	635.9	517.3

38	44.7	41.2	95.4	28.5	14.1	72	34.0	522.7	353.4
39	44.7	41.7	86.0	34.9	7.0	54	46.4	394.1	304.2
40	40.3	41.9	78.3	36.4	-2.5	40	63.9	308.4	221.3
41	49.3	41.0	78.9	30.1	-1.5	36	60.4	363.6	265.1
42	49.9	42.7	135.7	34.8	16.8	66	56.9	780.8	571.9
43	43.5	43.7	92.5	32.6	14.9	55	47.6	430.2	332.7
44	41.9	46.7	142.3	35.1	25.0	88	50.4	929.4	702.8
45	44.1	38.1	75.2	37.2	1.6	70	38.1	440.5	313.2
46	52.4	45.2	146.9	31.0	1.3	100	35.3	753.8	588.9
47	47.0	43.6	92.2	34.9	7.8	66	39.6	429.9	345.2
48	59.2	61.0	146.8	28.4	26.8	90	45.1	635.9	521.4
49	59.1	64.8	147.0	35.2	3.2	105	40.7	708.2	540.7
50	46.2	30.6	78.2	34.2	2.2	72	26.4	481.5	356.5
51	45.6	46.1	113.6	34.1	41.1	88	40.9	796.5	620.9
52	45.5	56.8	124.9	39.8	21.6	84	44.6	627.8	441.7
53	52.7	36.3	89.6	29.9	0.9	40	51.7	312.4	211.6
54	66.9	85.0	176.4	38.1	16.6	154	42.5	774.5	624.0
55	66.7	53.7	147.0	27.7	2.9	84	44.3	724.2	524.0
56	68.5	61.1	160.4	34.2	14.6	90	54.2	915.7	729.7
57	61.2	58.0	168.1	34.3	10.4	116	37.8	738.6	609.1
58	62.2	48.6	186.8	23.3	6.9	72	62.7	987.8	703.9
59	52.5	55.2	152.7	33.2	15.1	112	38.2	813.2	623.0
60	76.9	68.6	134.6	35.1	3.5	74	56.6	623.7	446.9
61	59.2	51.1	126.7	32.7	11.6	60	62.5	589.2	407.0
62	60.9	54.2	173.7	29.8	9.2	112	39.0	796.9	517.5
63	53.6	58.0	101.0	40.3	0.1	110	29.9	541.7	408.0
64	61.7	59.8	138.4	31.8	8.0	144	26.2	625.6	426.3
65	55.8	47.3	96.0	28.8	3.5	90	31.1	376.2	270.2
66	67.0	70.3	159.6	27.9	-0.3	120	35.0	665.8	512.8
67	72.3	63.8	168.4	31.9	5.9	84	60.7	844.8	646.2
68	62.4	50.6	165.2	26.4	5.4	126	30.2	812.5	589.2
69	67.0	58.0	126.8	33.3	5.7	78	48.4	474.0	376.3
70	71.6	60.7	174.7	31.6	16.2	64	81.9	898.0	703.1
71	59.1	59.7	165.0	33.9	7.9	144	33.2	851.3	636.8
72	57.5	47.3	90.2	31.3	2.5	46	56.6	437.5	323.8
73	59.3	53.9	154.5	27.9	14.8	126	32.3	670.0	474.4
74	67.9	48.1	95.5	36.8	10.8	52	56.5	398.7	298.4
75	60.6	49.1	172.2	24.5	6.7	84	48.3	561.3	397.3
76	43.7	37.8	117.5	37.0	7.5	57	62.5	842.6	542.7
77	51.3	48.9	150.8	27.1	9.0	78	53.2	823.2	603.8
78	50.3	59.4	129.6	43.6	9.0	104	43.5	779.3	555.1
79	63.2	65.9	155.5	36.4	9.7	168	25.5	694.7	550.0
80	59.4	64.3	139.4	38.9	10.8	120	36.5	694.8	495.0

81	63.3	54.5	162.3	32.6	11.6	84	56.9	809.3	589.5
82	47.6	47.4	85.2	38.8	9.4	69	38.5	459.7	370.6
83	57.7	61.9	155.1	36.2	6.5	108	44.6	731.7	466.4
84	60.5	64.9	153.5	44.4	10.6	128	37.3	801.4	607.1
85	72.1	62.2	147.6	37.2	7.9	140	32.4	801.4	592.7
86	58.8	42.4	123.3	36.7	0.9	40	92.0	721.6	450.8
87	57.6	31.2	101.8	25.1	3.5	48	55.8	547.9	416.8
88	65.9	64.1	120.5	31.9	4.4	81	43.6	406.5	312.9
