# Supplementary material for: Prediction of the Wingate anaerobic mechanical power outputs from a maximal incremental cardiopulmonary exercise stress test using machine-learning approach
Source: PLoS One. 2019 Mar 12;14(3):e0212199. doi: 10.1371/journal.pone.0212199 (PMC6413913; doi:10.1371/journal.pone.0212199)
Supplement: S3 Table — (PDF) [file pone.0212199.s003.pdf]

Table C in S3 File. Raw data of all features

Subject #	Max time	Max Rf	Max VT	Max VE	Max VO_2	Max VCO_2	Max VE/VO_2	Max VE/VCO_2	Max VO_2	Max HR	Max RR	VO_2 1st intersect	VO_2 2nd intersect	VCO_2 1st intersect	VCO_2 2nd intersect	V SLOP	Time to the VAT	Relative aerobic time	The anaerobic time	Relative time VAT	VAT ratio	Ratio of VAT to RR	Slope VCO_2 versus VE	Slop VO2 versus VE	Slope VO_2 versus time	Slope A	Slope B
1	660.0	62.5	2.6	156.7	4153.5	4738.2	37.7	35.8	57.7	190.0	1.1	2102.9	2232.6	1378.8	3652.6	2232.6	75.0	0.1	585.0	0.9	3.4	1957.1	29.9	0.043	3.8	0.4	1.6
2	615.0	43.0	3.0	126.4	3978.6	5361.0	31.8	25.3	58.8	183.0	1.3	1671.0	1903.0	1407.5	3799.0	1903.0	135.0	0.2	480.0	0.8	3.1	1412.3	42.8	0.035	4.8	0.8	1.7
3	705.0	49.9	2.8	131.9	3880.8	4818.7	34.5	36.0	54.1	188.0	1.2	1642.6	1731.0	998.6	3757.5	1731.0	60.0	0.1	645.0	0.9	2.5	1394.1	37.8	0.038	3.5	0.1	1.7
4	480.0	59.1	2.6	151.5	4025.2	4701.4	39.6	35.0	54.4	195.0	1.2	830.9	2377.4	847.5	1856.1	2377.4	105.0	0.2	375.0	0.8	5.0	2035.5	31.6	0.036	6.3	0.8	1.6
5	540.0	57.0	2.3	104.6	2875.3	3318.4	36.6	35.3	43.8	188.0	1.2	1065.1	2065.5	845.3	1845.0	2065.5	210.0	0.4	330.0	0.6	3.8	1778.3	32.9	0.037	3.8	0.8	1.8
6	525.0	55.2	1.7	90.9	2340.4	2696.8	39.1	40.1	34.9	195.0	1.2	1077.5	1804.6	826.1	1783.5	1804.6	345.0	0.7	180.0	0.3	3.4	1557.0	32.1	0.038	2.9	1.0	1.9
7	615.0	43.7	2.1	91.1	2302.7	2672.0	39.8	41.1	39.7	195.0	1.2	1005.5	1759.0	724.2	1706.2	1759.0	360.0	0.6	255.0	0.4	2.9	1507.1	32.1	0.038	2.9	1.0	2.1
8	510.0	51.7	1.6	80.9	2526.2	2774.6	34.1	40.3	48.6	197.0	1.1	647.7	1602.6	484.8	1296.0	1602.6	165.0	0.3	345.0	0.7	3.1	1459.1	35.9	0.034	3.0	0.7	1.6
9	435.0	43.2	2.3	92.2	2770.4	2967.5	40.9	37.3	41.3	173.0	1.2	1967.2	2157.2	1751.3	1837.3	2157.2	195.0	0.4	240.0	0.6	5.0	1871.7	34.9	0.030	4.9	0.8	1.8
10	585.0	46.2	2.1	96.2	3191.5	3295.9	48.1	36.8	49.1	187.0	1.3	833.0	1643.1	703.5	1327.3	1643.1	195.0	0.3	390.0	0.7	2.8	1257.4	34.5	0.029	4.9	0.7	1.3
11	495.0	35.6	1.9	54.7	1988.5	2092.9	29.5	34.8	38.2	190.0	1.1	1157.5	1332.3	836.2	1397.3	1332.3	180.0	0.4	315.0	0.6	2.7	1225.0	42.4	0.025	3.5	0.7	1.7
12	675.0	72.3	2.5	167.3	3983.0	4748.1	42.4	35.6	51.4	188.0	1.2	1819.2	3439.1	1498.3	3802.8	3439.1	480.0	0.7	195.0	0.3	5.1	2871.2	27.4	0.044	4.1	1.0	2.4
13	660.0	42.9	2.3	93.0	3135.6	3450.9	30.3	33.0	53.5	177.0	1.1	1476.0	1702.4	1042.4	2187.8	1702.4	150.0	0.2	510.0	0.8	2.6	1513.2	38.9	0.032	3.0	0.6	1.5
14	525.0	47.1	3.7	160.3	5339.6	6229.3	30.8	28.9	51.8	191.0	1.2	2390.4	2631.4	1978.9	5121.8	2631.4	60.0	0.1	465.0	0.9	5.0	2255.5	38.3	0.033	6.5	0.6	1.4
15	465.0	46.0	1.8	80.5	2600.0	2772.6	31.8	37.5	33.2	180.0	1.1	1235.4	1760.3	827.3	1321.4	1760.3	210.0	0.5	255.0	0.5	3.8	1617.1	36.8	0.032	4.6	0.7	2.0
16	675.0	49.1	2.7	123.1	3945.0	4570.5	31.6	27.7	55.5	198.0	1.2	1986.6	2780.1	1699.2	3031.0	2780.1	345.0	0.5	330.0	0.5	4.1	2399.6	35.7	0.036	3.4	0.8	1.8
17	495.0	39.4	2.3	82.2	2698.7	3034.4	36.4	41.0	43.2	178.0	1.2	1573.6	2131.3	1189.1	1919.1	2131.3	270.0	0.5	225.0	0.5	4.3	1831.8	41.4	0.029	3.6	0.9	2.0
18	555.0	50.3	1.9	83.8	2396.9	2733.7	35.0	33.9	44.4	188.0	1.2	1191.1	1490.2	927.3	1034.6	1490.2	120.0	0.2	435.0	0.8	2.7	1287.2	34.7	0.041	2.3	0.7	1.7
19	555.0	42.1	1.9	79.6	2887.2	2666.6	37.9	40.1	46.6	178.0	1.0	1193.2	1351.1	795.0	922.4	1351.1	165.0	0.3	390.0	0.7	2.4	1387.3	34.0	0.028	4.0	0.5	1.2
20	435.0	35.8	1.7	45.1	2128.9	1854.3	34.2	38.4	42.6	189.0	0.9	995.0	1534.4	700.1	1321.3	1534.4	195.0	0.4	240.0	0.6	3.5	1671.5	47.5	0.017	4.8	0.6	1.5
21	630.0	52.6	3.1	151.0	4007.9	5022.7	38.7	30.1	44.5	181.0	1.3	2423.3	3328.1	2441.2	3008.8	3328.1	360.0	0.6	270.0	0.4	5.3	2561.3	34.0	0.038	4.4	1.1	2.2
22	510.0	46.0	2.0	90.9	2544.3	3207.8	35.7	38.2	47.7	176.0	1.3	1217.1	1347.0	1018.3	2366.4	1347.0	90.0	0.2	420.0	0.8	2.6	1068.3	41.0	0.034	3.1	0.8	1.6
23	720.0	55.2	2.2	110.9	3029.7	3671.6	37.7	30.4	46.7	187.0	1.2	2018.1	2434.9	1946.6	1952.9	2434.9	390.0	0.5	330.0	0.5	3.4	1953.2	34.3	0.037	3.2	1.0	1.9
24	585.0	33.7	2.8	71.7	2486.3	2763.9	32.6	31.9	53.5	189.0	1.1	915.4	1006.1	703.2	1386.0	1006.1	150.0	0.3	435.0	0.7	1.7	897.6	41.3	0.028	3.6	0.5	1.3
25	660.0	46.2	1.9	79.9	2625.5	2976.7	31.0	32.4	41.4	183.0	1.1	1031.4	1828.6	756.0	1493.8	1828.6	240.0	0.4	420.0	0.6	2.8	1612.9	39.4	0.031	3.1	0.8	2.0
26	555.0	57.0	2.7	147.7	4414.4	4967.4	33.5	29.8	69.4	193.0	1.1	1939.8	3590.8	1217.6	3279.0	3590.8	360.0	0.6	195.0	0.4	6.5	3169.3	33.9	0.043	4.0	1.3	1.9
27	555.0	58.8	1.6	94.4	2943.1	3393.8	32.9	32.7	48.2	175.0	1.2	1612.5	1757.8	1187.3	2431.6	1757.8	135.0	0.2	420.0	0.8	3.2	1524.4	37.3	0.030	4.1	0.6	1.6
28	555.0	48.5	1.6	70.4	1924.5	2210.3	44.5	46.7	38.5	182.0	1.3	293.3	1100.0	252.5	554.9	1100.0	165.0	0.3	390.0	0.7	2.0	851.9	33.9	0.035	2.5	0.6	1.3
29	540.0	47.2	2.0	80.9	2534.5	2736.8	33.6	32.3	39.6	182.0	1.1	1468.3	1654.9	1159.5	1255.7	1654.9	165.0	0.3	375.0	0.7	3.1	1488.9	35.6	0.035	3.0	0.8	1.7
30	675.0	72.7	3.1	181.9	3628.8	4107.7	52.7	44.6	42.0	184.0	1.2	2079.4	2946.9	1698.1	1883.3	2946.9	390.0	0.6	285.0	0.4	4.4	2496.3	23.9	0.047	3.4	1.0	2.1
31	555.0	43.8	2.7	97.0	2773.1	3024.5	36.5	35.5	43.1	191.0	1.1	1327.5	1437.5	1025.1	2440.0	1437.5	150.0	0.3	405.0	0.7	2.6	1314.3	31.1	0.035	4.0	0.6	1.4
32	585.0	45.3	3.6	154.2	4396.1	5218.3	35.3	30.0	46.4	184.0	1.2	3029.3	3410.6	2515.7	4285.6	3410.6	360.0	0.6	225.0	0.4	5.8	2857.6	32.4	0.043	4.7	1.1	2.1
33	555.0	48.9	2.0	96.6	2171.5	2634.7	46.3	40.4	38.8	167.0	1.2	944.7	1698.7	760.2	1636.4	1698.7	255.0	0.5	300.0	0.5	3.1	1379.7	26.9	0.051	2.4	1.0	2.1
34	600.0	52.2	2.0	106.0	3346.6	3524.6	32.5	36.2	51.2	181.0	1.1	1566.2	2625.7	1114.0	2679.2	2625.7	405.0	0.7	195.0	0.3	4.4	2462.8	34.7	0.034	3.8	1.0	1.8
35	555.0	54.1	2.9	153.9	4172.4	4856.0	36.9	32.1	54.9	189.0	1.2	2788.8	3432.5	2127.4	3113.9	3432.5	315.0	0.6	240.0	0.4	6.2	2949.3	29.7	0.057	3.1	1.4	2.2
36	525.0	44.8	3.0	132.1	3311.4	3737.0	40.1	36.7	41.1	168.0	1.1	1647.5	1818.8	1207.3	2953.5	1818.8	45.0	0.1	480.0	0.9	3.5	1604.5	29.1	0.047	3.0	0.5	1.5
37	585.0	59.2	3.0	146.8	4055.2	4122.6	36.5	36.7	61.0	181.0	1.0	2187.7	3355.8	1604.2	3332.0	3355.8	330.0	0.6	255.0	0.4	5.7	3271.9	28.4	0.039	3.7	0.9	1.7
38	480.0	44.7	2.2	95.4	2447.7	2872.9	39.3	33.9	41.2	186.0	1.2	867.7	1932.5	752.1	1871.0	1932.5	255.0	0.5	225.0	0.5	4.0	1646.5	28.5	0.044	3.3	1.1	1.9
39	480.0	44.7	2.0	86.0	2503.1	2841.7	34.6	36.9	41.7	187.0	1.1	1128.4	1637.6	873.2	1547.4	1637.6	210.0	0.4	270.0	0.6	3.4	1442.5	34.9	0.035	3.7	0.7	1.9
40	435.0	40.3	2.0	78.3	2555.6	2719.3	31.1	36.0	41.9	185.0	1.1	1169.3	2069.4	869.0	2070.0	2069.4	255.0	0.6	180.0	0.4	4.8	1944.8	36.4	0.028	5.7	0.8	1.9
41	480.0	49.3	1.7	78.9	2175.1	2277.7	36.9	39.8	41.0	190.0	1.1	1019.7	1057.4	723.2	969.7	1057.4	120.0	0.3	360.0	0.8	2.2	992.9	30.1	0.036	3.9	0.6	1.4
42	495.0	49.9	2.9	135.7	3758.0	4362.6	36.8	32.9	42.7	182.0	1.2	3288.8	3350.6	2974.7	2994.1	3350.6	225.0	0.5	270.0	0.5	6.8	2832.0	34.8	0.045	3.1	1.1	2.3
43	360.0	43.5	2.3	92.5	2619.0	3092.8	35.7	31.5	43.7	194.0	1.2	1396.0	1900.0	1139.1	1815.6	1900.0	45.0	0.1	315.0	0.9	5.3	1593.4	32.6	0.047	2.4	0.4	1.7
44	615.0	41.9	3.5	142.3	4438.8	4915.2	32.1	31.4	46.7	182.0	1.1	2423.6	3463.6	1799.6	3498.0	3463.6	375.0	0.6	240.0	0.4	5.6	3127.9	35.1	0.037	4.5	1.0	1.8
45	435.0	44.1	1.8	75.2	2670.1	2858.1	29.6	29.0	38.1	178.0	1.1	1990.8	1905.3	1796.0	1806.2	1905.3	120.0	0.3	315.0	0.7	4.4	1763.8	37.2	0.039	2.4	1.0	1.5
46	660.0	52.4	2.8	146.9	3529.5	4543.1	41.6	32.3	45.2	186.0	1.3	2066.5	2216.2	1854.6	3184.6	2216.2	270.0	0.4	390.0	0.6	3.4	1721.8	31.0	0.043	4.5	0.9	1.9
47	585.0	47.0	2.0	92.2	2614.5	2978.4	38.5	36.7	43.6	185.0	1.1	1458.3	2086.3	1165.5	1569.7	2086.3	330.0	0.6	255.0	0.4	3.6	1831.4	34.9	0.033	3.3	0.9	2.1
48	585.0	59.2	3.0	146.8	4055.2	4122.6	36.5	36.7	61.0	183.0	1.0	2187.7	3355.8	1604.2	3332.0	3355.8	330.0	0.6	255.0	0.4	5.7	3271.9	28.4	0.039	3.7	0.9	1.7
49	615.0	59.1	2.5	147.0	4274.8	5270.9	34.4	30.5	64.8	174.0	1.2	2378.9	2423.0	2115.1	4458.7	2423.0	225.0	0.4	390.0	0.6	3.9	1965.1	35.2	0.033	5.8	0.8	1.6
50	570.0	46.2	1.8	78.2	1899.1	2487.0	42.6	33.1	30.6	182.0	1.3	964.9	1263.6	855.2	865.7	1263.6	210.0	0.4	360.0	0.6	2.2	942.0	34.2	0.040	2.4	0.8	2.1
51	555.0	45.6	2.9	113.6	3597.3	3819.7	31.7	37.4	46.1	193.0	1.1	1572.8	1724.8	992.8	2764.7	1724.8	45.0	0.1	510.0	0.9	3.1	1616.1	34.1	0.034	4.1	0.4	1.4
52	615.0	45.5	3.1	124.9	3749.3	5143.8	35.0	27.0	56.8	192.0	1.4	2165.9	2301.3	2027.4	3634.4	2301.3	75.0	0.1	540.0	0.9	3.7	1664.4	39.8	0.044	2.9	0.8	2.0
53	525.0	52.7	1.9	89.6	2066.8	2502.4	43.4	41.0	36.3	193.0	1.2	939.0	1317.2	747.2	1290.0	1317.2	225.0	0.4	300.0	0.6	2.5	1087.9	29.9	0.040	3.5	0.7	1.8
54	663.0	66.9	2.9	176.4	6543.0	6832.0	32.9	31.0	85.0	192.0	1.3	1375.7	2880.4	1420.1	2781.7	2782.8	111.0	0.2	552.0	0.8	4.2	2095.5	38.1	0.027	6.8	0.8	1.1
55	593.0	66.7	2.9	147.0	3723.0	4657.0	41.7	33.6	53.7	200.0	1.3	1493.6	1526.1	1105.4	1148.0	2118.8	15.0	0.0	578.0	1.0	3.6	1681.6	27.7	0.056	2.9	0.8	1.7
56	691.0	68.5	3.0	160.4	4878.0	5627.0	33.1	30.5	61.1	192.0	1.2	2908.4	3110.4	2348.4	2627.1	3298.8	233.0	0.3	458.0	0.7	4.8	2843.8	34.2	0.037	4.0	0.8	1.7
57	721.0	61.2	3.0	168.1	4379.0	5557.0	40.3	35.2	58.0	179.0	1.3	3227.7	3445.0	3013.4	3381.0	3658.1	285.0	0.4	436.0	0.6	5.1	2792.4	34.3	0.043	3.1	1.0	1.9
58	528.0	62.2	3.8	186.8	4511.0	5609.0	44.8	35.1	48.6	196.0	1.3	2647.7	2687.2	2203.8	2263.1	2500.0	44.0	0.1	484.0	0.9	4.7	1908.4	23.3	0.066	3.6	0.6	1.9
59	628.0	52.5	3.1	152.7	4275.0	4957.0	36.7	35.0	55.2	180.0	1.2	2259.0	2286.9	1738.1	1777.9	2508.2	64.0	0.1	564.0	0.9	4.0	2072.9	33.2	0.042	3.6	0.7	1.5
60	584.0	76.9	2.6	134.6	4192.0	4840.0	32.9	32.8	68.6	187.0	1.2	2753.4	2809.9	2442.0	2521.4	3105.0	29.0	0.0	555.0	1.0	5.3	2609.2	35.1	0.041	2.5	0.9	1.5
61	426.0	59.2	2.7	126.7	3750.0	4296.0	36.0	30.3	51.1	193.0	1.2	1984.0	2384.7	1659.6	2222.2	2309.0	53.0	0.1	373.0	0.9	5.4	1892.7	32.7	0.049	3.8	0.8	1.8
62	710.0	60.9	3.1	173.7	4373.0	5608.0	41.0	31.8	54.2	191.0	1.3	1902.0	3143.2	1647.6	3306.7	3132.6	359.0	0.5	351.0	0.5	4.4	2355.3	29.8	0.051	4.0	1.2	2.1
63	527.0	53.6	2.1	101.0	3289.0	4244.0	34.9	26.5	58.0	192.0	1.4	1385.3	1428.4	1229.0	1287.2	1845.8	82.0	0.2	445.0	0.8	3.5	1347.3	40.3	0.042	2.7	0.8	1.8
64	628.0	61.7	2.8	138.4	3777.0	4778.0	39.0	30.1	59.8	184.0	1.3	1320.9	1369.4	1063.5	1131.6	1800.0	48.0	0.1	580.0	0.9	2.9	1374.0	31.8	0.045	3.0	0.9	1.6
65	375.0	55.8	2.0	96.0	2801.0	3212.0	37.2	30.3	47.3	178.0	1.3	1466.5	1503.2	1220.0	1265.7	1900.0	5.0	0.0	370.0	1.0	5.1	1520.0	28.8	0.057	2.2	1.1	1.7
66	618.0	67.0	2.7	159.6	4203.0	4817.0	40.4	34.5	70.3	198.0	1.2	1667.7	2327.6	1571.3	2357.3	2148.6	62.0	0.1	556.0	0.9	3.5	1775.7	27.9	0.048	3.4	0.8	1.4
67	633.0	72.3	2.8	168.4	5101.0	5830.0	37.1	30.9	63.8	189.0	1.2	1891.6	2353.9	1626.0	2239.5	3200.0	227.0	0.4	406.0	0.6	5.1	2623.0	31.9	0.042	4.0	1.1	1.5
68	551.0	62.4	3.3	165.2	3808.0	4968.0	45.6	34.7	50.6	188.0	1.3	1670.3	2014.5	1340.9	1820.2	2110.4	61.0	0.1	490.0	0.9	3.8	1611.0	26.4	0.060	3.4	1.3	1.7
69	540.0	67.0	2.3	126.8	3777.0	4289.0	37.4	31.6	58.0	200.0	1.2	1477.9	1532.8	935.0	1009.4	2000.0	25.0	0.0	515.0	1.0	3.7	1626.0	33.3	0.048	3.1	0.5	1.6
70	674.0	71.6	3.2	174.7	5240.0	6144.0	36.3	32.1	60.7	184.0	1.2	1845.3	2205.2	1155.3	1613.3	2500.0	47.0	0.1	627.0	0.9	3.7	2100.8	31.6	0.044	4.0	0.8	1.5
71	680.0	59.1	3.2	165.0	4780.0	5818.0	35.4	28.8	59.7	181.0	1.2	2894.9	3607.3	2960.6	3911.2	3737.9	444.0	0.7	236.0	0.3	5.5	3014.4	33.9	0.039	4.3	1.2	1.8
72	420.0	57.5	1.9	90.2	2603.0	2833.0	36.2	32.6	47.3	187.0	1.1	1511.6	1520.7	1289.5	1301.3	2300.0	267.0	0.6	153.0	0.4	5.5	2017.5	31.3	0.047	2.4	1.4	1.3
73	579.0	59.3	2.9	154.5	4068.0	5041.0	39.4	32.5	53.9	182.0	1.3	1456.6	3390.2	925.2	3811.2	2919.0	240.0	0.4	339.0	0.6	5.0	2298.4	27.9	0.058	3.3	1.4	2.0
74	493.0	67.9	1.8	95.5	2939.0	3269.0	34.6	33.8	48.1	157.0	1.2	1315.8	1441.5	1011.2	1157.9	2300.0	274.0	0.6	219.0	0.4	4.7	1982.8	36.8	0.038	2.9	1.0	1.8
75	633.0	60.6	4.0	172.2	4060.0	4851.0	42.8	35.8	49.1	189.0	1.2	2209.9	3200.7	2000.0	3099.6	3169.6	283.0	0.4	350.0	0.6	5.0	2641.3	24.5	0.055	3.4	0.9	1.8
76	412.0	43.7	3.1	117.5	3561.0	4226.0	33.8	28.2	37.8	173.0	1.2	1753.6	2101.1	1405.7	1905.4	3100.0	289.0	0.7	123.0	0.3	7.5	2500.0	37.0	0.043	3.6	1.5	2.1
77	518.0	51.3	3.3	150.8	4151.0	4824.0	37.0	33.2	48.9	179.0	1.2	2014.6	3323.6	1532.8	3315.3	3198.5	216.0	0.4	302.0	0.6	6.2	2733.8	27.1	0.055	3.8	1.1	1.8
78	557.0	50.3	3.0	129.6	4524.0	5556.0	30.0	25.9	59.4	186.0	1.2	2991.3	3166.7	2632.2	2907.5	3346.6	237.0	0.4	320.0	0.6	6.0	2698.9	43.6	0.036	4.1	1.2	2.0
79	698.0	63.2	2.7	155.5	4289.0	5668.0	36.9	28.0	65.9	192.0	1.3	3229.0	3546.0	3450.5	3987.9	3536.8	503.0	0.7	195.0	0.3	5.1	2679.4	36.4	0.042	3.7	1.3	2.3
80	580.0	59.4	3.1	139.4	4377.0	5436.0	36.3	27.3	64.3	193.0	1.4	1377.6	1393.6	1388.6	1407.5	3500.0	349.0	0.6	231.0	0.4	6.0	2554.7	38.9	0.039	3.9	1.2	1.6
81	660.0	63.3	3.2	162.3	4782.0	5673.0	36.3	29.6	54.5	188.0	1.3	1904.8	2198.3	1281.7	1707.7	4000.0	447.0	0.7	213.0	0.3	6.1	3174.6	32.6	0.047	3.9	1.4	1.7
82	535.0	47.6	2.3	85.2	2659.0	3217.0	33.0	31.5	47.4	167.0	1.2	1819.6	1957.7	1608.3	1825.1	2076.9	165.0	0.3	370.0	0.7	3.9	1688.5	38.8	0.039	2.1	0.9	2.0
83	798.0	57.7	2.7	155.1	4817.0	5780.0	32.2	27.6	61.9	168.0	1.2	3349.1	4273.1	3095.5	4537.0	3859.8	546.0	0.7	252.0	0.3	4.8	3216.5	36.2	0.039	3.2	1.2	2.0
84	741.0	60.5	3.2	153.5	4774.0	6596.0	33.9	26.7	64.9	178.0	1.4	3369.6	3414.7	3573.6	3646.1	3929.3	499.0	0.7	242.0	0.3	5.3	2728.7	44.4	0.034	3.7	1.2	2.4
85	729.0	72.1	2.5	147.6	4534.0	5986.0	36.7	27.2	62.2	193.0	1.4	1593.0	2038.9	1651.8	2260.3	3600.0	486.0	0.7	243.0	0.3	4.9	2500.0	37.2	0.043	3.3	1.4	1.8
86	363.0	58.8	2.6	123.3	3679.8	4382.2	35.2	28.8	42.4	171.0	1.2	2240.4	2561.2	1899.3	2348.6	2968.2	124.0	0.3	239.0	0.7	8.2	2393.7	36.7	0.041	4.4	0.8	2.6
87	214.0	57.6	2.2	101.8	2679.3	2969.9	39.0	34.6	31.2	178.0	1.1	2210.0	2213.4	2179.0	2182.7	2200.0	7.0	0.0	207.0	1.0	10.3	1946.9	25.1	0.066	1.8	1.6	1.6
88	729.3	65.9	2.6	120.5	3527.9	3782.3	44.4	38.0	64.1	174.0	1.4	354.1	1377.6	150.2	1323.4	1250.0	109.1	0.1	620.3	0.9	1.7	899.3	31.9	0.039	2.5	0.7	1.5

Subject #	Slope RR versus time	Slope VT as a function of time	Area A	Area B	Slope 1st minute	Slope 2nd minute	Slope 3rd minute	VO_2 at 1st minute	VO_2 at 2nd minute	VO_2 at 3rd minute	Ratio of predicted VO2max/real VO2max	Ratio of predicted VO2 at VAT versus the real VO2	Time at the VAT	Rf at the VAT	VT at the VAT	VE at the VAT	VCO_2 at the VAT	VO_2 at the VAT	RR at AT	Max slope * max speed	VEmax/(max slope * speed)	VO2max/(max slope * max speed)	Predicted VO2 max	Max intensity
1	0.00063	0.00159	936364.0	5220025.8	22.2	13.5	8.4	1811.7	2384.6	2608.0	1.15	0.99	75.0	22.1	2.0	45.1	1479.5	33.0	0.6	130.0	1.2	31.9	66.3	85800.0
2	0.00077	0.00322	1238211.5	7032205.1	13.2	10.5	9.3	1315.8	1786.3	2175.0	0.88	0.99	135.0	30.0	1.4	43.3	1825.7	29.3	0.9	100.0	1.3	39.8	51.8	61500.0
3	0.00079	0.00194	227824.2	5567546.3	25.5	13.0	7.7	2017.8	1937.9	2175.9	1.23	1.03	60.0	21.2	1.5	31.7	1069.6	28.1	0.5	130.0	1.0	29.9	66.3	91650.0
4	0.00050	0.00339	1725136.5	4532065.8	17.3	22.7	15.7	1269.4	2644.7	2885.6	1.01	1.08	105.0	51.9	1.3	66.7	2191.0	37.0	0.8	99.0	1.5	40.7	55.0	47520.0
5	0.00075	0.00146	1178971.1	1369412.8	6.7	10.5	8.4	999.7	1797.8	1930.8	1.08	1.03	210.0	31.8	1.7	54.4	1926.2	35.1	0.8	70.0	1.5	41.1	47.3	37800.0
6	0.00065	0.00158	1001701.2	994580.2	9.4	7.3	4.7	1016.6	1407.9	1491.1	1.24	1.17	345.0	38.1	1.4	54.0	1723.0	27.7	0.9	88.0	1.0	26.6	43.4	46200.0
7	0.00063	0.00208	1027790.7	1124789.0	5.9	3.7	4.0	767.6	843.5	1234.9	1.18	1.18	360.0	31.3	1.7	53.1	1591.9	30.6	0.9	90.0	1.0	25.6	47.0	55350.0
8	0.00081	0.00162	744778.8	1719797.1	20.1	10.9	6.4	1336.1	1588.3	1609.9	1.12	1.26	165.0	34.5	1.1	39.4	1275.5	31.7	0.8	72.0	1.1	35.1	54.3	36720.0
9	0.00022	0.00223	1596268.8	1211149.4	11.7	5.8	6.8	1178.4	1196.7	1964.0	1.04	0.95	195.0	31.8	1.9	60.0	1997.7	34.2	0.9	40.0	2.3	69.3	42.8	17400.0
10	0.00008	0.00174	557237.8	3402879.0	-9.7	2.3	4.3	235.8	851.9	1399.7	1.35	1.68	195.0	40.9	1.0	42.0	1357.4	25.9	0.8	130.0	0.7	24.5	66.3	76050.0
11	0.00065	0.00221	547038.9	810289.5	4.9	7.0	7.1	508.1	922.0	1375.3	1.04	1.23	180.0	21.1	1.4	30.5	1075.0	26.4	0.8	64.0	0.9	31.1	39.8	31680.0
12	0.00042	0.00188	4176646.9	1692592.7	16.8	12.9	8.7	1627.0	2035.3	2256.5	1.20	1.10	480.0	52.8	2.1	108.9	3593.9	46.1	1.0	120.0	1.4	33.2	61.5	81000.0
13	0.00059	0.00106	707354.8	2614969.0	18.0	7.7	5.3	1373.9	1497.9	1779.0	1.06	0.99	150.0	22.4	1.7	38.6	1405.5	33.0	0.7	110.0	0.8	28.5	56.7	72600.0
14	0.00057	0.00351	1731599.1	10013214.6	49.7	21.0	12.4	3006.8	3044.9	3258.6	1.20	1.24	60.0	27.4	2.2	61.7	2400.6	29.2	0.8	104.0	1.5	51.3	62.4	54600.0
15	0.00098	0.00280	818801.2	1189101.8	7.5	6.3	6.6	827.2	1138.1	1708.6	1.13	1.21	210.0	36.7	1.4	49.6	1609.2	26.9	0.8	48.0	1.7	54.2	37.4	22320.0
16	0.00042	0.00153	2376238.1	3815606.8	22.0	11.5	6.6	2104.5	2300.5	2340.9	1.11	1.17	345.0	32.1	2.0	63.4	2540.5	40.2	0.9	120.0	1.0	32.9	61.5	81000.0
17	0.00074	0.00241	1494494.4	642793.2	20.9	12.7	8.0	1424.7	1766.1	1945.6	1.12	1.20	270.0	26.7	2.0	53.2	2078.8	36.3	0.9	55.0	1.5	49.1	48.4	27225.0
18	0.00080	0.00198	408260.5	533873.1	9.2	6.4	4.5	1204.5	1588.8	1572.3	1.13	0.98	120.0	43.6	0.9	40.3	1269.0	29.4	0.8	66.0	1.3	36.3	50.1	36630.0
19	0.00035	0.00208	332158.5	2072322.3	-2.0	6.0	6.6	270.7	936.1	1213.0	1.02	1.38	165.0	28.9	1.2	34.4	981.8	23.5	0.7	70.0	1.1	41.2	47.3	38850.0
20	0.00013	0.00197	501898.7	626573.4	1.8	4.7	6.2	414.6	881.1	1222.9	1.08	1.13	195.0	21.2	1.3	28.4	1028.4	32.1	0.6	60.0	0.8	35.5	45.8	26100.0
21	0.00052	0.00281	4505935.0	2362833.7	9.6	10.2	9.5	1507.1	2156.8	2468.9	1.27	1.17	360.0	40.1	2.5	101.6	3646.2	37.0	1.1	110.0	1.4	36.4	56.7	69300.0
22	0.00080	0.00237	553628.1	2290385.3	15.4	8.6	5.6	1294.5	1470.2	1721.5	1.05	1.14	90.0	40.3	1.0	39.3	1269.7	28.5	0.8	90.0	1.0	28.3	50.3	45900.0
23	0.00048	0.00147	1998132.9	1372749.3	6.7	4.1	4.7	1060.7	1171.6	1673.2	1.06	1.02	390.0	37.5	1.9	72.2	2597.0	38.2	1.0	95.0	1.2	31.9	49.4	68400.0
24	0.00057	0.00277	184050.9	2529830.3	2.7	4.4	4.1	658.2	932.6	1245.6	0.89	1.31	150.0	20.1	1.0	20.7	766.7	22.1	0.7	70.0	1.0	35.5	47.3	40950.0
25	0.00073	0.00167	1048209.5	1473446.1	6.2	4.6	4.3	792.3	988.2	1336.0	1.07	1.03	240.0	29.6	1.4	41.1	1612.0	31.7	0.8	72.0	1.1	36.5	44.3	47520.0
26	0.00075	0.00210	3282005.4	1501740.3	18.2	5.6	3.4	2750.3	2679.7	2817.9	0.96	0.95	360.0	44.2	2.1	94.5	3299.9	57.0	0.9	112.0	1.3	39.4	67.0	62160.0
27	0.00052	0.00150	1046934.2	2635889.3	1.5	13.3	10.7	638.8	1716.5	1980.2	1.04	1.22	135.0	36.7	1.0	37.4	1355.5	29.7	0.7	66.0	1.4	44.6	50.1	36630.0
28	0.00092	0.00197	373423.9	111004.4	-2.0	5.0	6.0	256.5	829.6	1109.1	1.08	1.30	165.0	30.3	1.0	29.2	886.2	22.3	0.8	54.0	1.3	35.6	41.6	29970.0
29	0.00074	0.00210	693763.3	914450.4	6.9	5.3	5.8	1064.4	1402.4	1735.8	1.12	1.10	165.0	30.0	1.3	39.4	1359.4	26.3	0.8	72.0	1.1	35.2	44.3	38880.0
30	0.00053	0.00190	3225760.8	1370420.9	16.9	9.3	6.9	1687.8	1890.6	2087.6	1.48	1.36	390.0	41.2	2.2	89.5	2800.6	34.6	0.9	104.0	1.7	34.9	62.4	70200.0
31	0.00049	0.00255	499885.8	2527261.6	5.5	6.1	6.4	840.5	1124.5	1621.0	1.10	1.37	150.0	28.0	1.2	32.8	1125.1	23.7	0.7	70.0	1.4	39.6	47.3	38850.0
32	0.00081	0.00276	3453982.5	3450932.6	18.4	14.9	10.0	1867.2	2381.6	2808.3	1.25	1.27	360.0	29.8	3.0	88.5	3231.0	37.1	0.9	96.0	1.6	45.8	57.9	56160.0
33	0.00080	0.00179	686879.5	282938.8	9.3	7.3	5.5	965.1	1283.8	1498.6	1.35	1.23	255.0	41.9	1.4	59.5	1684.1	32.3	0.9	60.0	1.6	36.2	52.5	33300.0
34	0.00059	0.00173	2370499.4	1835549.1	18.6	9.0	6.3	1478.9	1607.8	1846.5	1.07	1.12	405.0	46.3	1.6	73.2	2395.4	41.7	0.9	99.0	1.1	33.8	55.0	59400.0
35	0.00087	0.00212	2028027.8	2714920.4	6.0	0.8	2.3	2749.3	2708.5	2946.1	1.15	1.11	315.0	41.9	2.1	88.0	3171.1	45.8	0.9	132.0	1.2	31.6	63.3	73260.0
36	0.00065	0.00225	480928.3	3150797.9	16.1	7.6	5.1	1987.3	2291.1	2456.3	1.19	1.16	45.0	27.1	1.8	48.6	1444.6	25.0	0.7	80.0	1.7	41.4	48.8	42000.0
37	0.00038	0.00273	3825454.7	2150655.4	26.8	12.3	8.1	2353.5	2649.8	2934.5	1.10	1.00	330.0	41.9	2.3	94.6	2945.3	50.9	0.9	90.0	1.6	45.1	67.0	52650.0
38	0.00068	0.00188	1052552.7	1081876.6	14.1	5.4	4.7	1230.3	1285.4	1601.5	1.08	0.97	255.0	30.6	1.8	53.6	1868.9	33.6	0.9	72.0	1.3	34.0	44.3	34560.0
39	0.00083	0.00273	765550.8	1392804.8	7.0	4.5	5.4	933.7	1119.5	1618.4	1.00	1.02	210.0	35.8	1.3	47.7	1472.1	31.9	0.8	54.0	1.6	46.4	41.6	25920.0
40	0.00049	0.00347	1555164.8	737871.9	-2.5	5.0	7.2	413.8	995.4	1512.4	1.02	0.99	255.0	34.8	1.8	61.9	1946.7	36.7	0.9	40.0	2.0	63.9	42.8	17400.0
41	0.00064	0.00267	185620.3	1602477.1	-1.5	6.5	6.7	399.7	1149.2	1346.9	0.95	1.29	120.0	33.3	0.9	28.6	802.3	21.7	0.7	36.0	2.2	60.4	38.9	17280.0
42	0.00083	0.00267	2534506.6	913452.8	16.8	6.3	5.1	2725.1	2814.5	3043.9	1.17	1.12	225.0	35.8	2.6	92.5	3126.5	38.8	0.9	66.0	2.1	56.9	50.1	32670.0
43	0.00097	0.00272	354110.8	1382511.7	14.9	5.8	3.5	1853.3	1901.2	2179.0	1.11	1.06	45.0	36.5	1.3	49.2	1686.9	34.3	0.8	55.0	1.7	47.6	48.4	19800.0
44	0.00060	0.00271	4093885.7	3085107.5	25.0	8.5	6.5	2324.8	2112.1	2691.0	1.14	1.15	375.0	26.8	3.2	85.2	3170.0	37.1	0.9	88.0	1.6	50.4	53.4	54120.0
45	0.00068	0.00099	182867.0	1605562.2	1.6	0.9	1.7	1800.6	1929.9	2049.2	1.24	1.31	120.0	29.7	1.4	41.0	1547.7	27.6	0.8	70.0	1.1	38.1	47.3	30450.0
46	0.00070	0.00236	1262677.8	3746208.1	1.3	4.9	4.1	899.5	1314.7	1522.0	1.15	1.26	270.0	38.8	1.4	55.3	2011.7	28.8	0.9	100.0	1.5	35.3	51.8	66000.0
47	0.00050	0.00188	1533164.0	1243819.9	7.8	7.7	7.2	892.0	1314.8	1615.7	1.15	1.11	330.0	32.8	1.6	53.8	1955.6	35.1	0.9	66.0	1.4	39.6	50.1	38610.0
48	0.00038	0.00273	3825454.7	2150655.4	26.8	12.3	8.1	2353.5	2649.8	2934.5	1.10	1.00	330.0	41.9	2.3	94.6	2945.3	50.9	0.9	90.0	1.6	45.1	67.0	52650.0
49	0.00033	0.00251	2526076.1	6554906.9	3.2	17.5	15.0	532.3	2169.4	2230.9	1.07	1.35	225.0	36.8	1.6	58.9	2262.6	37.6	0.9	105.0	1.4	40.7	69.3	64575.0
50	0.00090	0.00186	526872.8	582778.7	2.2	3.0	4.0	651.7	837.2	1243.5	1.45	1.46	210.0	24.7	1.4	34.8	1242.1	22.3	0.9	72.0	1.1	26.4	44.3	41040.0
51	0.00067	0.00248	97046.7	4086857.3	41.1	18.3	10.0	2015.8	2237.1	2310.2	1.16	1.28	45.0	19.3	1.8	34.0	1033.7	22.6	0.6	88.0	1.3	40.9	53.4	48840.0
52	0.00071	0.00248	1683853.4	4337981.6	21.6	10.3	6.5	2292.5	2577.2	2668.8	0.99	0.88	75.0	30.3	1.8	53.4	2295.4	37.1	0.9	84.0	1.5	44.6	56.1	51660.0
53	0.00072	0.00206	590817.3	720036.4	0.9	4.1	5.9	449.9	772.5	1294.7	1.18	1.33	225.0	28.2	1.4	40.2	1233.2	24.4	0.9	40.0	2.2	51.7	42.8	21000.0
54	0.00002	0.00215	2341120.5	15957955.0	16.6	12.9	9.8	1552.0	2535.0	2674.0	0.74	0.82	113.0	23.7	2.6	61.3	2346.0	37.0	0.8	154.0	1.1	42.5	63.3	102102.0
55	0.00076	0.00186	134068.0	2890783.5	2.9	3.9	3.4	2081.0	2353.0	2533.0	1.17	0.98	0.0	25.5	1.9	47.4	1766.0	30.7	0.8	84.0	1.8	44.3	62.8	49812.0
56	0.00057	0.00115	3078552.5	3487897.0	14.6	13.3	9.4	2296.0	2795.0	2853.0	1.10	0.88	235.0	25.5	2.3	59.8	2559.0	42.0	0.8	90.0	1.8	54.2	67.0	62190.0
57	0.00057	0.00214	3024323.5	918601.0	10.4	6.8	5.1	2191.0	1922.0	3191.0	1.19	0.89	286.0	46.8	2.0	92.7	3202.0	49.1	0.9	116.0	1.4	37.8	69.2	83636.0
58	0.00086	0.00136	562298.5	4639102.5	6.9	7.2	5.6	2693.0	3006.0	3141.0	1.12	1.11	48.0	15.6	3.4	53.1	2129.0	27.1	0.9	72.0	2.6	62.7	54.3	38016.0
59	0.00060	0.00241	1386310.5	2786977.5	15.1	9.3	6.6	2235.0	2764.0	2765.0	1.21	1.03	66.0	29.7	1.9	57.9	1953.0	32.6	0.8	112.0	1.4	38.2	67.0	70336.0
60	0.00046	0.00120	1977202.5	1038364.5	3.5	2.1	2.3	2734.0	2868.0	2928.0	1.11	0.82	30.0	54.1	1.3	71.4	2678.0	53.3	0.8	74.0	1.8	56.6	76.3	43216.0
61	0.00090	0.00231	905413.5	2559364.5	11.6	8.9	5.9	2309.0	2469.0	2780.0	0.90	0.84	55.0	26.6	2.0	53.1	2007.0	31.8	0.9	60.0	2.1	62.5	45.8	25560.0
62	0.00065	0.00203	3624684.0	504783.5	9.2	10.2	7.4	1858.0	2263.0	2400.0	1.24	1.12	361.0	43.7	2.2	95.6	3404.0	41.7	1.0	112.0	1.6	39.0	67.0	79520.0
63	0.00085	0.00155	212826.0	1760676.5	0.1	1.2	2.0	1739.0	2096.0	2216.0	0.98	0.91	84.0	26.0	1.5	39.1	1792.0	36.9	0.9	110.0	0.9	29.9	56.7	57970.0
64	0.00052	0.00105	689814.5	3857462.0	8.0	6.2	4.6	1945.0	2230.0	2430.0	1.09	1.02	51.0	22.3	1.7	38.3	1585.0	29.5	0.9	144.0	1.0	26.2	65.1	90432.0
65	0.00090	0.00028	251440.5	488381.5	3.5	2.2	2.3	1973.0	1966.0	2235.0	0.99	0.86	0.0	24.1	1.7	40.0	1667.0	34.9	0.8	90.0	1.1	31.1	47.0	33750.0
66	0.00039	0.00210	321532.0	2603204.5	-0.3	3.1	3.1	1758.0	2084.0	2104.0	0.87	0.83	64.0	45.0	1.4	60.9	2058.0	36.5	0.9	120.0	1.3	35.0	61.5	74160.0
67	0.00039	0.00159	1956554.5	3735374.5	5.9	4.6	3.9	2458.0	2640.0	2845.0	0.98	0.88	228.0	44.6	1.9	85.6	3218.0	41.7	1.0	84.0	2.0	60.7	62.8	53172.0
68	0.00076	0.00039	771118.0	1031665.0	5.4	5.5	4.9	2078.0	2476.0	2483.0	1.13	1.16	64.0	20.4	2.6	53.1	2011.0	28.8	0.9	126.0	1.3	30.2	57.4	69426.0
69	0.00084	0.00227	607118.0	428349.5	5.7	3.0	2.8	1940.0	2092.0	2426.0	1.01	0.98	26.0	43.2	0.9	39.9	1333.0	30.8	0.7	78.0	1.6	48.4	58.5	42120.0
70	0.00059	0.00081	1136388.0	6710012.5	16.2	9.2	6.8	2557.0	2931.0	2975.0	1.09	0.97	50.0	22.2	2.3	50.0	1991.0	31.0	0.7	64.0	2.7	81.9	66.4	43136.0
71	0.00038	0.00233	5062630.0	2253262.5	7.9	7.8	5.7	1942.0	2414.0	2413.0	1.09	1.08	446.0	39.9	2.6	103.7	3900.0	47.1	1.0	144.0	1.1	33.2	65.1	97920.0
72	0.00066	0.00133	1131947.0	663014.0	2.5	2.8	2.6	1611.0	1794.0	1947.0	1.03	0.95	268.0	42.1	1.7	70.5	2359.0	42.2	1.0	46.0	2.0	56.6	48.7	19320.0
73	0.00075	0.00196	2684498.0	3142351.0	14.8	8.6	5.5	2367.0	2601.0	2694.0	1.06	0.75	242.0	34.0	2.3	79.3	3024.0	40.4	1.0	126.0	1.2	32.3	57.4	72954.0
74	0.00073	0.00113	1414867.5	1028988.5	10.8	5.3	3.9	1785.0	1661.0	1969.0	1.14	1.04	275.0	39.5	1.6	61.2	2110.0	38.6	0.9	52.0	1.8	56.5	54.6	25636.0
75	0.00047	-0.00038	3273804.0	1532047.5	6.7	9.7	8.1	1900.0	2536.0	2721.0	1.28	1.04	286.0	23.3	3.3	77.4	2987.0	38.6	0.9	84.0	2.1	48.3	62.8	53172.0
76	0.00077	0.00179	2575583.0	70539.5	7.5	5.6	4.4	2133.0	2477.0	2688.0	1.16	1.10	291.0	31.5	2.8	89.7	3346.0	34.7	1.0	57.0	2.1	62.5	43.7	23484.0
77	0.00069	0.00202	2301918.5	2794770.5	9.0	6.4	4.5	2561.0	2776.0	2971.0	1.20	1.07	218.0	30.6	2.6	79.7	2910.0	37.7	0.9	78.0	1.9	53.2	58.5	40404.0
78	0.00080	0.00149	3069536.0	2992929.5	9.0	6.9	5.5	2481.0	2759.0	3118.0	1.05	0.98	238.0	29.0	2.5	71.8	3181.0	44.4	0.9	104.0	1.2	43.5	62.4	57928.0
79	0.00058	0.00168	4492707.5	3087657.0	9.7	4.2	3.4	1987.0	2144.0	2380.0	1.04	1.04	504.0	44.0	2.3	100.7	3889.0	55.8	1.1	168.0	0.9	25.5	68.7	117264.0
80	0.00063	0.00212	2280467.5	900904.5	10.8	5.6	4.3	2385.0	2357.0	2337.0	0.96	0.88	350.0	37.3	2.5	92.3	4010.0	53.7	1.1	120.0	1.2	36.5	61.5	69600.0
81	0.00072	0.00102	4131490.0	1351834.0	11.6	6.6	4.9	2308.0	2617.0	2769.0	1.15	1.07	448.0	47.8	2.5	120.2	4374.0	46.7	1.1	84.0	1.9	56.9	62.8	55440.0
82	0.00065	0.00068	868357.5	980105.0	9.4	4.8	3.6	1757.0	1837.0	1885.0	1.10	0.87	167.0	29.2	1.7	50.3	1950.0	38.3	0.9	69.0	1.2	38.5	52.2	36915.0
83	0.00042	0.00146	4446512.0	4282406.5	6.5	2.5	2.7	2418.0	2405.0	2682.0	1.29	1.19	567.0	41.9	2.4	99.2	3931.0	50.4	1.0	108.0	1.4	44.6	79.7	86184.0
84	0.00043	0.00139	5975325.5	589876.5	10.6	5.9	4.0	2360.0	2476.0	2682.0	1.17	1.06	501.0	36.7	2.7	100.8	4446.0	53.6	1.1	128.0	1.2	37.3	76.0	94848.0
85	0.00047	0.00114	3131809.0	2415261.0	7.9	6.4	4.1	1999.0	2390.0	2226.0	1.14	1.09	487.0	54.4	2.1	113.0	4278.0	49.8	1.2	140.0	1.1	32.4	71.2	102060.0
86	0.00115	0.00055	1619296.5	1308936.6	0.9	5.7	7.1	2089.1	2726.9	3121.1	1.01	0.98	126.0	25.5	2.4	62.4	2559.6	34.4	0.9	40.0	3.1	92.0	42.8	14520.0
87	0.00071	-0.00106	193586.4	398708.1	3.5	2.8	2.4	2323.8	2471.3	2649.9	1.20	1.04	9.0	35.2	2.0	69.7	2171.8	25.9	1.0	48.0	2.1	55.8	37.4	10272.0
88	0.00077	0.00127	472735.0	1496134.4	4.4	6.6	6.5	896.7	1025.5	1680.6	0.95	1.15	112.1	27.0	1.1	29.1	986.3	23.3	0.8	81.0	1.5	43.6	60.7	59076.3

Subject #	Slope 1st minute VE	VCO_2 at 1 minute	Rf at 1 minute	HR at 1 minute	VO_2 at 1 min	VE at 1 minute	Slope 2 minute VE	VCO_2 at 2 min	Rf at 2 min	HR at 2 min	VO_2 at 2 min	VE at 2 min	Slope 3 minute VE	VCO_2 at 3 min	Rf at 3 min	HR at 3 min	VO_2 at 3 min	VE at 3 min	PP [w/kg]	MP [w/kg]
1	0.2	1209.5	21.0	103.3	25.2	38.3	0.2	1810.5	28.8	131.3	33.1	56.3	0.2	2063.7	26.6	138.8	36.2	61.0	11.2	8.1
2	0.2	1129.3	23.8	86.5	19.4	27.0	0.2	1678.9	23.0	107.3	26.4	37.9	0.2	2230.2	30.2	118.0	32.1	51.2	11.3	7.8
3	0.2	1069.6	21.2	105.8	28.1	31.7	0.2	1357.2	21.4	118.8	27.0	35.6	0.2	1831.3	20.8	125.3	30.3	47.9	12.2	8.5
4	0.8	1437.0	52.0	135.5	17.2	50.3	0.5	2299.3	53.1	165.8	35.7	69.6	0.4	2796.7	55.2	171.8	39.0	86.4	9.3	6.7
5	0.0	729.7	18.6	89.3	15.2	21.8	0.2	1352.2	31.7	127.5	27.4	44.4	0.2	1680.9	25.1	147.5	29.4	47.8	8.1	6.5
6	0.2	755.3	33.2	105.8	15.2	28.4	0.1	1053.4	29.1	119.8	21.0	34.3	0.1	1228.9	37.1	135.3	22.3	40.6	7.1	5.1
7	0.1	569.1	25.7	108.3	13.2	22.9	0.1	622.5	25.4	123.3	14.5	23.2	0.1	1030.9	27.4	136.3	21.3	38.2	8.2	6.2
8	0.4	869.5	34.3	110.2	25.7	31.5	0.2	1187.5	33.6	140.5	30.5	38.3	0.1	1275.7	38.2	153.0	31.0	39.2	6.9	5.0
9	0.2	1048.3	29.1	115.8	17.6	34.7	0.1	1130.5	31.8	131.0	17.9	37.7	0.1	1682.8	34.7	142.3	29.3	52.7	7.1	4.7
10	-0.2	279.4	12.7	109.3	3.6	9.8	0.0	700.5	19.7	136.0	13.1	21.6	0.1	1136.7	44.6	144.5	21.5	36.7	11.9	8.9
11	0.1	416.1	17.8	100.5	9.8	13.5	0.1	677.9	17.3	133.3	17.7	21.4	0.1	1075.0	21.1	153.0	26.4	30.5	7.6	5.2
12	0.3	1331.1	34.2	91.5	21.0	37.9	0.2	1801.6	30.6	107.3	26.3	49.2	0.2	2054.5	34.4	111.3	29.1	55.0	10.7	8.6
13	0.3	985.9	18.2	124.0	23.4	29.0	0.1	1085.1	22.4	135.0	25.6	30.2	0.1	1332.6	21.1	137.0	30.4	35.0	9.4	7.1
14	0.8	2400.6	27.4	117.8	29.2	61.7	0.4	2827.7	29.4	142.8	29.6	70.5	0.3	3245.5	33.5	151.8	31.6	83.6	11.5	8.3
15	0.1	628.0	22.8	99.8	10.6	21.6	0.1	736.4	28.9	120.5	14.5	25.4	0.1	1200.4	36.7	138.3	21.8	38.3	6.4	4.6
16	0.2	1655.6	21.2	123.3	29.6	41.1	0.1	1905.2	27.8	143.5	32.4	47.9	0.1	1815.3	26.8	148.0	32.9	43.5	11.3	8.6
17	0.4	1071.6	24.1	113.3	22.8	34.1	0.2	1460.9	26.2	136.5	28.3	41.1	0.2	1701.4	25.6	148.0	31.1	47.1	10.1	7.7
18	0.2	901.0	41.2	102.3	22.3	29.9	0.1	1269.0	43.6	120.8	29.4	40.3	0.1	1410.8	41.2	140.5	29.1	44.3	8.7	6.4
19	-0.1	205.6	13.9	95.5	4.4	7.9	0.1	657.7	33.4	111.8	15.1	26.1	0.1	871.1	32.0	124.0	19.6	31.2	6.9	5.6
20	0.1	369.3	19.5	115.3	8.3	14.2	0.1	591.8	21.8	138.5	17.6	19.8	0.1	799.6	24.0	157.3	24.5	23.9	9.5	6.6
21	0.2	1482.4	29.6	95.8	16.7	42.0	0.2	2104.7	35.0	122.3	24.0	58.9	0.3	2582.4	35.4	134.5	27.4	70.9	11.0	8.7
22	0.4	1045.3	44.7	114.3	24.3	36.0	0.2	1352.1	41.0	143.0	27.6	40.9	0.1	1668.9	36.3	152.0	32.3	45.0	7.7	6.0
23	0.1	1026.3	20.7	101.0	16.3	28.1	0.1	1138.2	19.6	110.5	18.1	29.6	0.1	1523.8	19.3	125.3	25.8	37.2	9.7	7.0
24	0.0	519.4	20.3	86.5	14.2	15.8	0.0	745.6	12.4	95.8	20.1	19.8	0.0	1019.7	13.0	115.8	26.8	25.3	9.0	6.6
25	0.1	618.7	16.8	93.0	12.5	18.0	0.1	737.4	22.7	103.0	15.6	21.8	0.1	1031.9	25.1	118.0	21.1	28.7	7.5	5.7
26	0.3	1917.6	30.5	124.5	43.2	51.8	0.2	2172.4	31.1	150.8	42.1	59.6	0.2	2307.7	36.5	156.0	44.3	65.0	10.4	8.1
27	0.1	634.2	23.2	113.0	10.5	18.9	0.2	1264.9	30.8	137.0	28.1	34.7	0.2	1607.7	39.4	147.5	32.5	43.2	8.6	6.8
28	-0.1	222.1	25.8	93.4	5.1	10.2	0.1	536.9	30.6	109.9	16.6	20.1	0.1	954.9	33.9	128.3	22.2	30.6	5.9	4.3
29	0.1	766.3	30.9	101.3	16.6	23.1	0.1	1104.7	28.5	124.5	21.9	32.6	0.1	1434.1	35.2	136.0	27.1	42.2	7.0	5.2
30	0.3	1352.3	25.2	104.8	19.6	44.1	0.2	1645.6	16.8	123.8	21.9	49.2	0.2	1684.0	27.7	129.8	24.2	53.9	10.4	8.1
31	0.2	768.8	17.7	90.8	13.1	23.7	0.1	894.5	19.1	108.5	17.5	25.6	0.1	1416.6	22.8	137.3	25.2	38.7	7.8	5.8
32	0.2	1206.0	14.0	109.8	19.7	30.1	0.4	1949.3	31.9	131.5	25.1	55.5	0.3	2409.9	28.8	138.8	29.7	67.1	9.0	7.2
33	0.1	709.0	19.5	96.3	17.2	24.5	0.2	1046.1	29.6	115.5	22.9	36.0	0.2	1421.0	33.3	127.3	26.8	50.3	6.7	5.1
34	0.3	1015.1	23.6	93.8	22.6	31.3	0.2	1164.1	33.1	113.3	24.6	37.0	0.2	1469.1	37.2	116.8	28.3	47.5	9.5	7.9
35	0.4	2090.7	31.1	112.3	36.2	58.9	0.1	2094.8	31.9	136.8	35.6	56.8	0.1	2403.0	36.0	146.3	38.8	67.2	11.4	8.7
36	0.3	1482.5	28.9	97.5	24.7	49.2	0.2	1939.7	29.9	121.5	28.5	62.4	0.2	2190.7	30.4	129.3	30.5	68.9	11.9	8.9
37	0.4	1791.8	38.1	123.0	35.4	57.0	0.2	2150.4	39.8	147.3	39.8	66.8	0.2	2452.1	39.2	153.5	44.1	75.8	9.6	7.8
38	0.3	1012.1	24.3	106.5	20.7	30.8	0.1	1149.5	22.7	128.0	21.6	33.2	0.2	1563.9	21.3	143.8	27.0	42.8	9.0	6.0
39	0.2	740.4	27.8	105.0	15.6	26.6	0.1	821.6	28.6	133.3	18.7	28.6	0.1	1243.0	32.6	155.0	27.0	41.4	6.7	5.1
40	0.0	354.1	23.8	98.3	6.8	12.6	0.1	723.6	23.0	124.0	16.3	23.4	0.2	1206.3	25.0	145.8	24.8	37.4	5.1	3.6
41	0.0	366.8	28.7	90.8	7.5	14.6	0.1	802.3	33.3	125.5	21.7	28.6	0.2	1103.8	31.0	156.8	25.4	35.9	6.9	5.0
42	0.3	2154.5	36.5	122.5	31.0	66.4	0.2	2336.8	33.6	141.3	32.0	71.2	0.1	2677.7	36.0	153.0	34.6	80.3	8.9	6.5
43	0.4	1751.6	31.1	129.0	30.9	50.4	0.2	1949.0	34.5	155.0	31.7	54.6	0.1	2207.2	35.4	165.8	36.3	60.6	7.2	5.5
44	0.3	1675.7	21.6	94.5	24.4	47.8	0.2	1621.4	19.6	113.8	22.2	46.0	0.2	2166.0	28.2	124.3	28.3	64.6	9.8	7.4
45	0.1	1466.2	29.9	110.5	25.7	41.0	0.0	1547.7	29.7	129.5	27.6	41.0	0.1	1839.4	35.1	138.5	29.3	50.8	6.3	4.5
46	0.0	794.2	15.7	84.3	11.5	20.4	0.1	1089.8	18.7	95.8	16.8	27.7	0.1	1239.5	18.6	107.5	19.5	30.4	9.7	7.5
47	0.1	738.6	23.9	81.5	14.9	24.5	0.1	1109.0	25.7	108.5	21.9	33.1	0.1	1404.9	28.7	126.3	26.9	39.8	7.2	5.8
48	0.4	1791.8	38.1	142.0	35.4	57.0	0.2	2150.4	39.8	149.0	39.8	66.8	0.2	2452.1	39.2	155.0	44.1	75.8	9.6	7.8
49	0.1	581.5	21.1	113.0	8.1	17.3	0.4	1882.8	38.9	137.5	32.9	53.5	0.3	1961.3	34.4	144.3	33.8	51.4	10.9	8.3
50	0.0	543.3	20.3	103.0	10.5	16.4	0.1	682.7	22.0	125.8	13.5	21.2	0.1	1071.0	25.8	150.0	20.1	31.8	7.6	5.8
51	0.7	1190.9	24.1	115.3	25.8	36.3	0.5	1821.5	32.7	141.5	28.7	54.5	0.3	2041.4	29.1	152.5	29.6	60.6	10.3	8.0
52	0.3	2158.3	24.2	128.0	34.7	49.5	0.2	2703.8	34.7	148.3	39.0	63.2	0.2	3061.9	28.0	152.8	40.4	67.4	9.5	6.7
53	0.1	439.8	27.6	105.0	7.9	18.0	0.1	579.3	21.1	127.5	13.6	20.4	0.1	1041.6	28.5	140.8	22.7	35.1	5.5	3.7
54	0.4	1493.0	29.9	111.0	20.2	38.7	0.2	2283.0	37.0	128.0	32.9	55.6	0.2	2621.0	39.2	126.0	34.7	66.8	10.1	8.1
55	0.1	1738.0	32.3	127.0	30.0	47.4	0.1	2077.0	39.6	143.0	34.0	56.9	0.1	2375.0	30.1	157.0	36.6	62.5	10.4	7.6
56	0.1	1697.0	30.5	106.0	28.8	47.8	0.2	2051.0	29.5	115.0	35.0	54.6	0.2	2359.0	36.1	132.0	35.8	63.9	11.5	9.1
57	0.2	1664.0	34.5	107.0	29.0	46.7	0.2	1676.0	33.6	120.0	25.5	48.9	0.1	2566.0	42.3	128.0	42.3	73.1	9.8	8.1
58	0.0	2220.0	21.4	137.0	29.0	55.4	0.1	2658.0	23.9	148.0	32.4	65.6	0.1	2945.0	24.9	156.0	33.8	70.1	10.6	7.6
59	0.3	1718.0	32.3	128.0	28.9	53.2	0.2	2329.0	32.1	141.0	35.7	68.6	0.2	2449.0	32.9	152.0	35.7	71.4	10.5	8.0
60	0.0	2418.0	60.0	147.0	44.7	67.9	0.0	2549.0	62.5	152.0	46.9	72.4	0.1	2708.0	40.0	157.0	47.9	72.9	10.2	7.3
61	0.3	1976.0	34.6	150.0	31.5	53.8	0.3	2242.0	38.6	165.0	33.6	61.2	0.2	2695.0	34.0	172.0	37.9	69.2	8.2	5.7
62	0.2	1604.0	24.8	92.0	23.0	43.0	0.2	2022.0	27.3	108.0	28.0	51.3	0.2	2361.0	32.6	113.0	29.7	63.8	10.0	6.5
63	0.1	1574.0	29.5	120.0	30.7	37.6	0.0	1942.0	29.3	119.0	37.0	43.1	0.0	2098.0	29.8	132.0	39.1	47.2	9.6	7.2
64	0.0	1751.0	20.5	128.0	30.8	41.8	0.1	2186.0	23.3	142.0	35.3	54.0	0.1	2472.0	34.3	154.0	38.5	65.0	9.9	6.8
65	0.2	1916.0	25.8	142.0	33.3	50.6	0.2	1946.0	37.3	151.0	33.2	54.0	0.1	2294.0	46.2	157.0	37.8	67.6	6.3	4.5
66	0.0	1626.0	41.4	135.0	29.4	47.9	0.1	2015.0	49.8	144.0	34.8	62.1	0.1	2066.0	46.7	157.0	35.2	61.9	11.4	8.8
67	0.2	2404.0	32.8	132.0	30.7	63.6	0.2	2660.0	32.2	143.0	33.0	69.4	0.1	2814.0	39.3	147.0	35.6	77.0	10.7	8.2
68	0.2	1905.0	18.5	118.0	27.6	50.8	0.2	2456.0	22.0	135.0	32.9	65.8	0.2	2614.0	26.5	137.0	33.0	71.9	10.4	7.6
69	0.2	1412.0	43.5	137.0	29.8	42.6	0.1	1788.0	27.3	154.0	32.1	47.3	0.1	2131.0	43.3	164.0	37.3	61.4	7.3	5.8
70	0.3	1973.0	23.3	123.0	29.6	51.3	0.2	2376.0	26.3	131.0	34.0	60.3	0.2	2550.0	22.7	139.0	34.5	62.1	10.4	8.2
71	0.2	1918.0	27.2	125.0	24.2	48.6	0.2	2516.0	35.3	141.0	30.1	67.0	0.2	2395.0	26.2	147.0	30.1	58.4	10.7	8.0
72	0.1	1445.0	38.8	130.0	29.3	46.2	0.1	1699.0	40.4	148.0	32.6	53.3	0.1	1879.0	39.3	158.0	35.4	57.2	7.9	5.9
73	0.3	1959.0	32.0	73.0	31.4	51.6	0.3	2429.0	35.4	77.0	34.5	63.3	0.2	2639.0	37.9	80.0	35.7	69.1	8.9	6.3
74	0.2	1429.0	31.7	119.0	29.2	45.6	0.1	1366.0	40.3	121.0	27.2	43.3	0.1	1739.0	38.1	120.0	32.2	53.8	6.6	4.9
75	0.1	1837.0	13.9	141.0	23.0	45.4	0.2	2205.0	14.9	152.0	30.7	54.0	0.2	2610.0	20.9	159.0	32.9	67.2	6.7	4.8
76	0.2	1909.0	23.4	126.0	22.6	50.3	0.2	2447.0	25.4	139.0	26.3	68.0	0.2	2787.0	29.3	148.0	28.5	76.7	8.9	5.7
77	0.2	2203.0	23.3	133.0	30.2	59.2	0.1	2467.0	27.7	143.0	32.7	68.0	0.1	2635.0	28.5	147.0	35.0	71.5	9.7	7.1
78	0.1	1983.0	24.2	118.0	32.6	47.5	0.2	2378.0	29.6	129.0	36.2	57.3	0.1	2810.0	28.5	139.0	40.9	64.3	10.2	7.3
79	0.2	1817.0	28.1	120.0	30.5	46.8	0.1	2060.0	28.8	123.0	32.9	52.9	0.1	2299.0	32.7	130.0	36.6	59.1	10.7	8.5
80	0.1	2406.0	26.7	136.0	35.0	55.2	0.2	2520.0	34.7	144.0	34.6	61.7	0.1	2407.0	40.8	149.0	34.3	60.7	10.2	7.3
81	0.2	1787.0	19.0	128.0	26.3	45.1	0.1	2109.0	24.3	134.0	29.8	55.5	0.1	2281.0	28.2	140.0	31.6	57.5	9.3	6.8
82	0.1	1518.0	29.2	127.0	31.3	41.6	0.1	1739.0	34.1	131.0	32.7	48.7	0.1	1796.0	28.6	142.0	33.6	47.0	8.2	6.6
83	0.3	2105.0	37.3	101.0	31.1	58.1	0.1	2058.0	36.6	104.0	30.9	55.7	0.1	2320.0	35.1	111.0	34.5	59.2	9.4	6.0
84	0.2	2379.0	29.5	110.0	32.1	59.2	0.2	2681.0	32.2	116.0	33.6	67.9	0.1	2899.0	34.7	119.0	36.4	73.7	11.2	8.5
85	0.1	2081.0	29.8	119.0	27.4	49.4	0.1	2640.0	35.9	130.0	32.8	63.7	0.1	2381.0	37.3	135.0	30.5	57.2	11.0	8.1
86	0.0	1818.0	24.4	130.0	24.1	49.4	0.1	2293.3	22.4	137.0	31.5	55.0	0.1	2887.3	29.0	156.0	36.0	71.4	8.5	5.3
87	0.1	2336.3	41.8	164.0	27.1	75.8	0.2	2594.8	51.7	171.0	28.8	86.9	0.2	2919.3	56.9	175.0	30.9	99.0	6.4	4.9
88	0.0	591.1	18.4	75.0	16.3	17.4	0.1	820.4	24.6	92.0	18.6	24.6	0.2	1429.7	32.3	112.0	30.6	41.1	7.4	5.7
